# Scaffolding Protein GspB/OutB Facilitates Assembly of the Dickeya dadantii Type 2 Secretion System by Anchoring the Outer Membrane Secretin Pore to the Inner Membrane and to the Peptidoglycan Cell Wall

**DOI:** 10.1128/mbio.00253-22

**Published:** 2022-05-12

**Authors:** Shiheng Zhang, Shuang Gu, Piers Rycroft, Florence Ruaudel, Frederic Delolme, Xavier Robert, Lionel Ballut, Richard W. Pickersgill, Vladimir E. Shevchik

**Affiliations:** a Université Lyon, Université Lyon 1, INSA Lyon, CNRS UMR 5240 Microbiologie Adaptation et Pathogénie, Villeurbanne, France; b School of Biological and Chemical Sciences, Queen Mary University of London, London, United Kingdom; c Protein Science Facility, SFR BioSciences, CNRS, UMS3444, Lyon, France; d Université Lyon, Molecular Microbiology and Structural Biochemistry, UMR5086 CNRS, Lyon, France; Duke University School of Medicine

**Keywords:** membrane proteins, *Dickeya dadantii*, pathogenic bacteria, peptidoglycan, scaffolding protein GspB, secretin GspD, type 2 secretion system

## Abstract

The phytopathogenic proteobacterium Dickeya dadantii secretes an array of plant cell wall-degrading enzymes and other virulence factors via the type 2 secretion system (T2SS). T2SSs are widespread among important plant, animal, and human bacterial pathogens. This multiprotein complex spans the double membrane cell envelope and secretes fully folded proteins through a large outer membrane pore formed by 15 subunits of the secretin GspD. Secretins are also found in the type 3 secretion system and the type 4 pili. Usually, specialized lipoproteins termed pilotins assist the targeting and assembly of secretins into the outer membrane. Here, we show that in *D. dadantii*, the pilotin acts in concert with the scaffolding protein GspB. Deletion of *gspB* profoundly impacts secretin assembly, pectinase secretion, and virulence. Structural studies reveal that GspB possesses a conserved periplasmic homology region domain that interacts directly with the N-terminal secretin domain. Site-specific photo-cross-linking unravels molecular details of the GspB-GspD complex *in vivo*. We show that GspB facilitates outer membrane targeting and assembly of the secretin pores and anchors them to the inner membrane while the C-terminal extension of GspB provides a scaffold for the secretin channel in the peptidoglycan cell wall. Phylogenetic analysis shows that in other bacteria, GspB homologs vary in length and domain composition and act in concert with either a cognate ATPase GspA or the pilotin GspS.

## INTRODUCTION

Gram-negative bacteria are surrounded by two membranes, the inner membrane (IM) and the outer membrane (OM), that together delimit a thin (~30-nm) periplasmic space containing the peptidoglycan (PG) layer (1, 2). To exchange proteins, nucleic acids, and sugars with the external medium, these bacteria have evolved an array of specialized transport systems (3–7). The type 2 secretion system (T2SS) is widespread in proteobacteria and secretes folded proteins that play a pivotal role in colonization of different niches, survival, competition, and pathogenicity (8–12). The plant-pathogenic gammaproteobacterium Dickeya dadantii uses the T2SS, called Out, to secrete pectinases in infected plant tissues, causing soft rot disease in numerous plants and root vegetables (13, 14). The T2SS is embedded in both the IM and the OM and spans the entire cell envelope of the bacteria. It is composed of 12 core components, generically called GspC to GspM and GspO (OutC to OutO in *D. dadantii*) as well as some additional components, GspA, -B, -N, and -S, that are present in certain T2SSs (8–10, 15, 16).

The secretin is an essential T2SS component that forms gated channels in the OM, through which the effector proteins are translocated in the medium or at the cell surface. The secretins are also shared by the type 3 secretion system (T3SS), type 4 pili (T4P), and the competence and the filamentous phage assembly systems (16–19). Secretin homologs have also been identified in mitochondria of some eukaryotes (20). Recent cryo-electron microscopy (cryo-EM) structures have revealed important molecular details of the assembly of the megadalton-sized secretin channels consisting of 12 to 16 subunits, with a clear predominance of 15-fold symmetry in more recently acquired near-atomic-resolution structures of the T2SS and T3SS secretins (15, 21–28). Only a small apical segment of the secretin channel, consisting of an amphipathic helical loop and a β-lip, is embedded in the OM, while the main portion, which is composed of the core secretin domain together with the N-terminal domains, N0 to N3, is located in the periplasm. The conserved C domains of 15 secretin subunits together constitute a double-β-barrel channel composed of 60 internal and 60 external β-strands. Domains N1 to N3, also termed ring-building motifs, adopt a mixed α/β fold and together form a cylinder like structure protruding through the periplasm. In the T2SS secretins, the N-terminal N0 domain forms the first gate at the entry of the secretin channel that interacts with the IM portion of the secretion machinery and controls the recruitment of substrates (29–33). This portion of the secretin channel is not seen in the majority of reported structures or appears as smeared density, consistent with its flexibility (21, 23, 34). However, in the recent high-resolution cryo-EM structure of Klebsiella pneumoniae PulD, the N0 and N1 domains were resolved as a tightly packed ring, stabilized by interaction with the inner membrane PulC component (27). The molecular details of this interaction remain elusive, but this study shows the relevance of attachment of the secretin channel to the periplasmic portion of the IM components.

Usually, a small lipoprotein called pilotin guides the cognate secretin subunits through the periplasm to the OM and facilitates their assembly (16, 35). Two groups of pilotins with dissimilar sequences and structures have been identified in various T2SSs. The paradigm pilotins of the OutS/PulS family from *D. dadantii*, K. pneumoniae, and enterohemorrhagic Escherichia coli bind a short C-terminal region of secretin, termed the S domain, and pilot the secretin subunit via the Lol system to the inner leaflet of the outer membrane (36–39). Upon binding of pilotin, the disordered S domain folds into an α-helix (40–42). In the assembled secretin channel, the bound pilotins stabilize the external β-barrel by stapling tandemly arranged secretin subunits (15, 43). The T2SS pilotins of the AspS/ExeS family that were identified in *Vibrio*, *Aeromonas*, and enterotoxigenic and enteropathogenic E. coli seem to fulfil exactly the same functions as OutS/PulS while adopting a profoundly different fold (15, 43–45). Targeting and assembly of secretins from the T3SS and T4P systems are also facilitated by specific piloting lipoproteins with different structures and modes of action. For instance, the T3SS pilotins MxiM and ExsB are structurally not related to one another, to PilF/PilW family pilotins from the T4P system, or to the T2SS pilotins (46–49).

Beside the pilotins, some other assistance proteins are also involved in the assembly of the secretin channels through the bacterial cell wall. The main portion of the secretin channel is located in the periplasm (18 to 20 nm), and consequently, it crosses through and could interact with the PG mesh (27, 34). Indeed, the distance between the OM and the PG layer is 12 to 25 nm and is controlled by the size of the major OM lipoprotein Lpp, or Braun’s lipoprotein, which covalently attaches the PG to the OM (1, 50). In high-resolution cryo-EM tomography of the Salmonella enterica serovar Typhimurium T3SS and the Myxococcus xanthus T4P, the PG layer was visualized around the N-terminal portion of the respective secretin channels (25, 51). In the M. xanthus T4P, the N0 domain of the secretin PilQ is preceded by a triplet of specialized PG-binding AMIN domains (25, 52). In addition, another T4P component, TsaP, carries a LysM-like PG-binding domain; together, they provide anchoring of the secretin channel to the PG (53). The PG-binding modules have been identified in the other transenvelope machineries of Gram-negative bacteria, illustrating that anchoring to the PG is essential for their assembly and function (54–56).

In the T2SS, only LspD secretin from *Legionella* is known to possess a specialized PG-binding SPOR domain, located at the N terminus just prior to the N0 domain (34). Another known example of a PG-binding T2SS component is the multimodular ATPase GspA, which possesses a periplasmic PG-binding domain of the Pfam family PF01471 (57). GspA has been identified in *Vibrio*, *Aeromonas*, and some other bacteria, where it acts together with GspB and is thought to be implicated in the insertion of the secretin into or through the PG mesh (58, 59). GspB is also present in some other *Enterobacteriaceae*, such as *Dickeya*, Klebsiella, and *Pectobacterium*, but in this case without a GspA counterpart (60). Previous studies have suggested that GspB could interact with the secretin, but its precise role has remained unclear, and reports regarding GspB functions are conflicting (60–63).

In this study, we explored the structure and function of OutB/GspB from the plant pathogen *D. dadantii*; we reveal that the GspB periplasmic domain is structurally similar to the homology region (HR) of GspC and depict the molecular details of its interaction with the N0 domain of the secretin. We demonstrate that OutB/GspB guides the secretin to the outer membrane and anchors the secretin to the inner membrane and to the cell wall peptidoglycan.

## RESULTS

### OutB is required for type 2 secretion and essential for the full virulence of *D. dadantii*.

**FIG 1:**
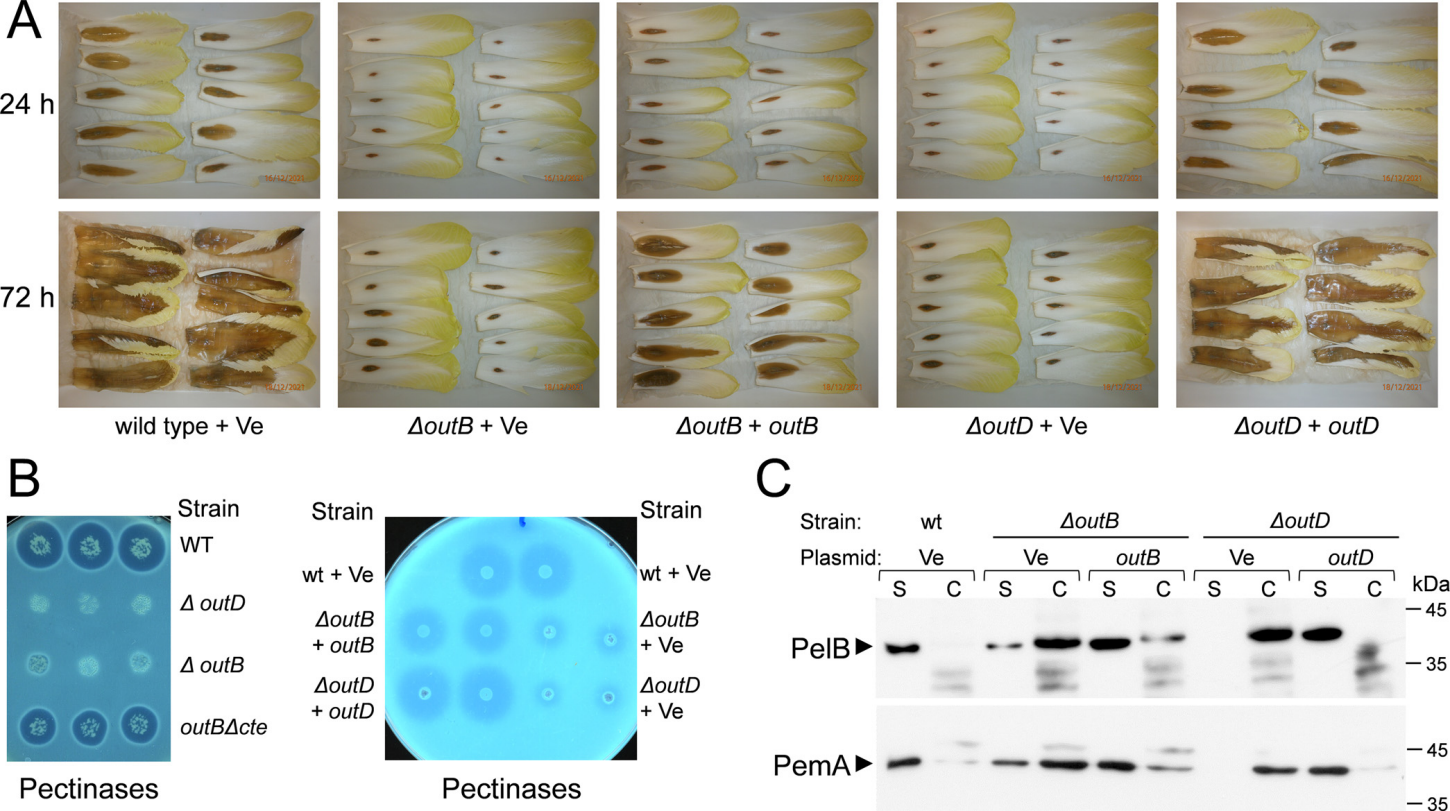
OutB is required for the type 2 secretion and full virulence of *D. dadantii*. (A) Pathogenicity tests with chicory leaves. Chicory leaves were inoculated with 10^7^ cells of *D. dadantii* wild-type, Δ*outB*, or Δ*outD* strains (*D. dadantii* A5652, A5654, and A6533, respectively) carrying either an empty pGM-T vector (Ve) or a plasmid expressing the *outB* or *outD* gene and incubated at 28°C for the indicated time. (B) Pectinase plate secretion assay. *D. dadantii* strains carrying the same mutations and plasmids as in panel A were grown for 14 h at 30°C on plates containing polygalacturonate and then flooded with copper acetate. Halo size reflects the level of pectinase secretion. (C) Immunoblotting secretion assay. *D. dadantii* strains carrying the same mutations and plasmids as in panels A and B were grown for 14 h at 28°C on LB broth supplemented with galacturonate. Then, culture supernatants (S) and cells (C) were separated by SDS-PAGE and probed with antibodies raised against pectate lyase (PelB) and pectin methylesterase (PemA). The ratio of the pectinases in the S fraction reflects the efficiency of secretion. Of note is that the observed virulence and secretion defects in both Δ*outB* and Δ*outD* strains were efficiently complemented with the plasmids expressing either the *outB* or *outD* gene.

The collective action of several pectinases secreted by *D. dadantii* Out T2SS causes soft-rot maceration of infected plants, while mutant bacteria lacking functional Out system are fully or partially avirulent (14, 64). Therefore, to examine the functional relevance of OutB, we assessed its importance in the context of plant infection. Pathogenicity assays with chicory leaves clearly showed that the *D. dadantii* Δ*outB* mutant was barely virulent in comparison with the wild-type strain. A very small rotted area formed by the Δ*outB* strain 24 h postinfection did not progress further and was surrounded by dehydrated necrotic plant tissues that formed a barrier to bacterial proliferation (Fig. 1A). *D. dadantii* Δ*outD*, which lacks the secretin pore and was used as a negative control, provoked very similar symptoms. Since soft-rot symptoms result from the action of pectinases secreted by the Out system, these data indicate that Δ*outB* and Δ*outD* strains were both unable to efficiently secrete pectinases into the plant tissue. Consistent with this, in a plate secretion assay that shows pectin degradation by the Out-secreted enzymes, the Δ*outB* strain generated no or a very small halo, similar to that of the Δ*outD* strain (Fig. 1B). Western blotting with antibodies raised against the pectate lyase PelB and pectin methylesterase PemA confirmed that the Δ*outB* strain is not able to efficiently secrete these pectinases (Fig. 1C). These data show a loss or malfunction of the Out T2SS in the Δ*outB* strain that causes a striking reduction of bacterial virulence *in planta*.

### OutB is attached to both the inner and outer membranes.

**FIG 2:**
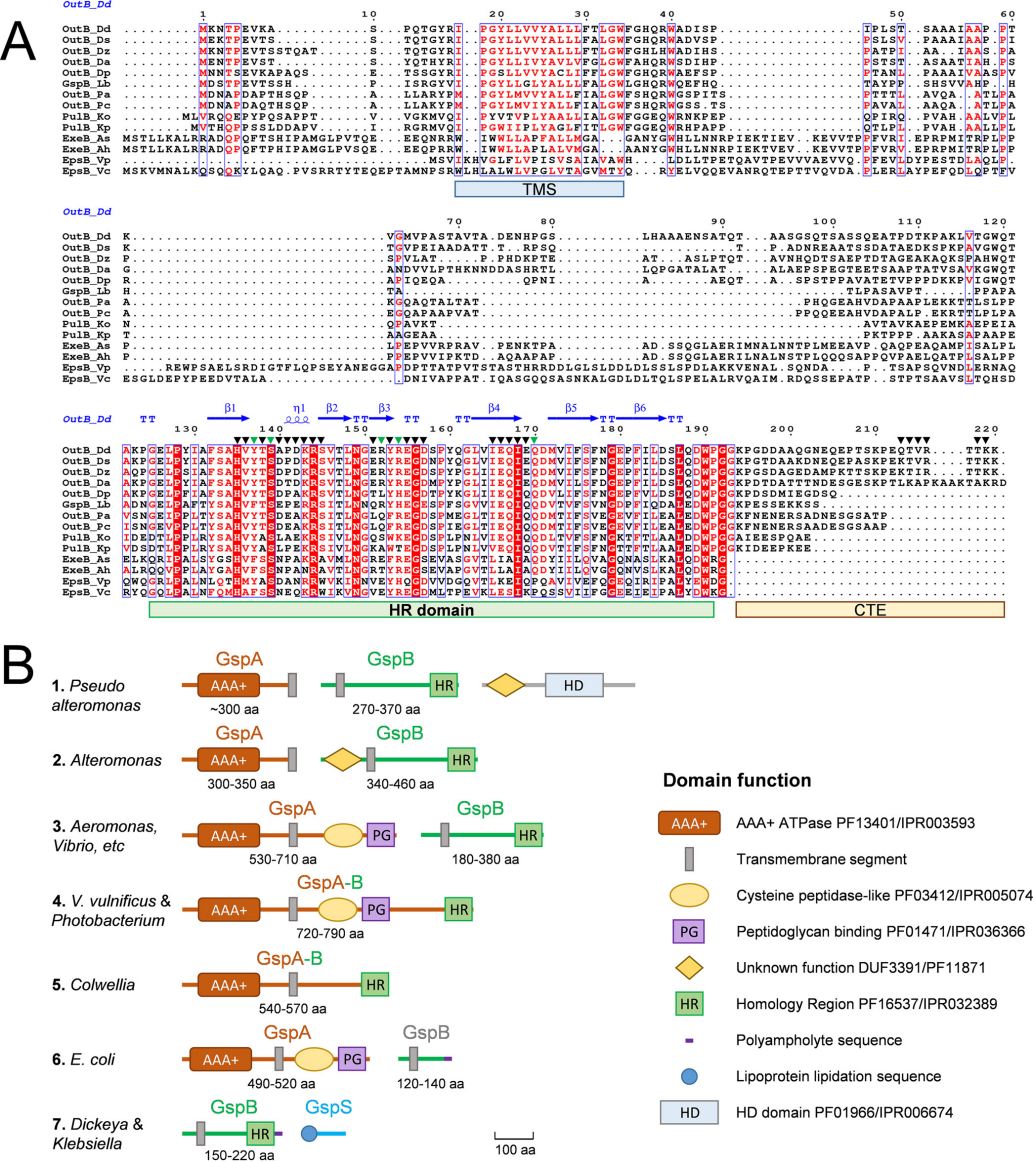
GspB organization. (A) Alignment of representative GspB sequences. Multiple-sequence alignment was performed with Clustal Omega (ebi.ac.uk) and the ESPript server (107). The secondary structure elements are shown for the OutB HR domain (PDB entry 4WFW). The residue numbering is that for *D. dadantii* OutB. The groups of identical and similar residues are indicated with red highlighting and red font, respectively. The residues substituted with *p*BPA and used in photo-cross-linking are shown with triangles, with green triangles indicating the residues generating an abundant complex with OutD. Positions of the transmembrane segment (TMS), the homology region (HR), and the C-terminal extension (CTE) are indicated with colored bars. Accession numbers of the protein sequences used are listed in Table S2. (B) Typical gene and domain organizations of GspB and related GspA and GspS proteins. For each GspB representative shown in Fig. S8A, gene synteny and domain organization were analyzed with the NCBI (www.ncbi.nlm.nih.gov), InterPro (www.ebi.ac.uk/interpro), and Pfam (pfam.xfam.org) databases and summarized into seven archetypes, named according to the most abundant or most studied representative bacteria. The protein size range (in amino acids) is indicated for each group. Of note is that more than 80% of analyzed GspB proteins belong to archetype 3.

**FIG 3:**
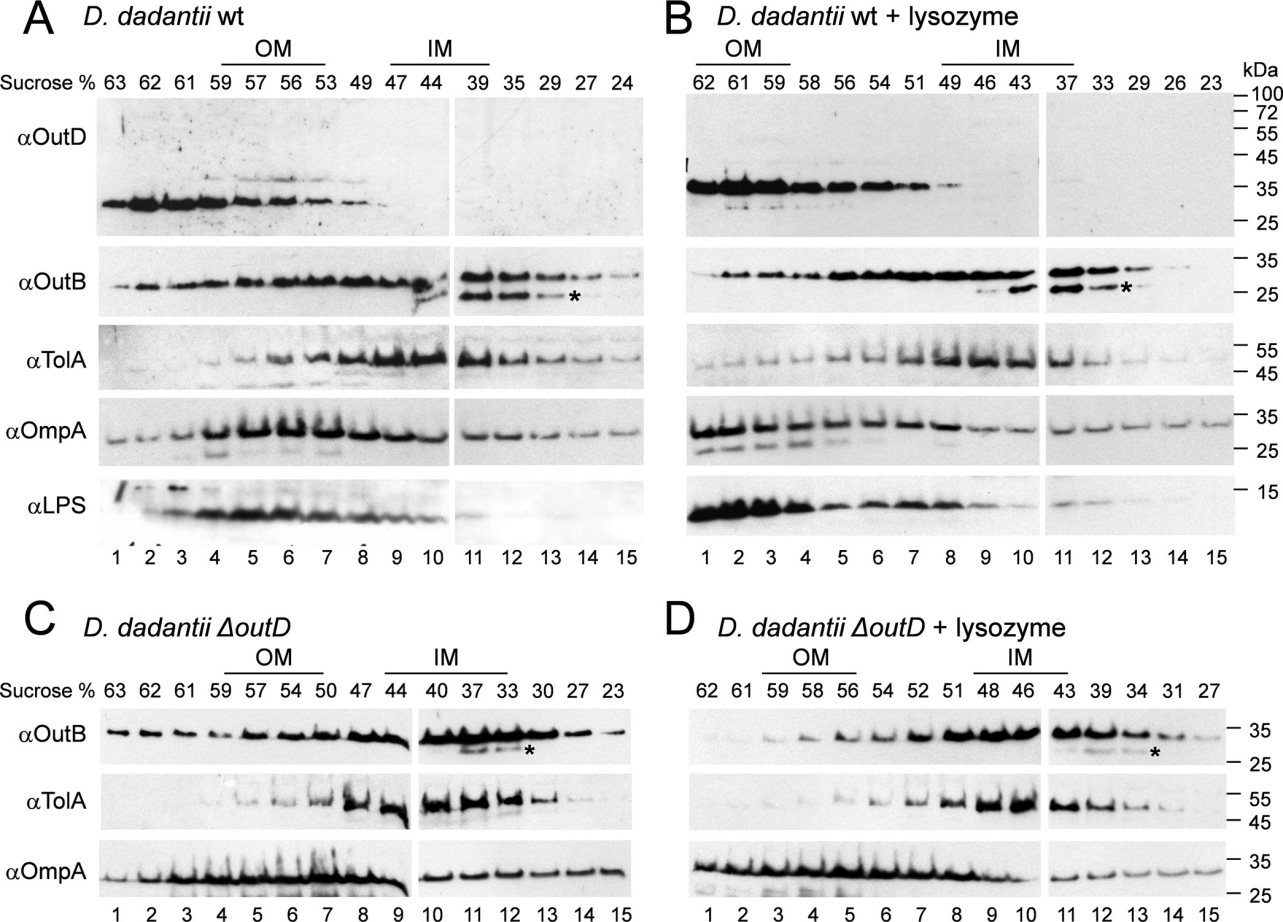
OutB is associated with both the inner and the outer membranes. *D. dadantii* A5652 wt (A and B) and A5653 *outD* (C and D) cells were grown on pectin-containing plates, broken with a French pressure cell, and membranes were separated on sucrose gradient and analyzed by immunoblotting with the indicated antibodies. For panels B and D, the broken cell extracts were treated with lysozyme prior loading onto the gradient. The positions of the inner and the outer membrane fractions are indicated according to the positions of LPS and OmpA (OM) as well as TolA (IM). A degradative product of OutB is indicated with an asterisk.

To examine if the absence of OutB compromises the integrity of the T2SS, the quantity of various T2SS components was assessed by immunoblotting (Fig. S1A). The abundance of the inner membrane assembly platform component OutC and the major pseudopilin OutG did not vary significantly, while the quantity of the secretin OutD decreased in the *D. dadantii* Δ*outB*. Conversely, the amount of OutB was obviously lower in *D. dadantii* Δ*outD* (Fig. S1A). These data indicate that OutB is required for secretin channel biogenesis or stability. OutD is localized in the OM, while OutB is predicted to reside in the IM due to its hydrophobic N-terminal segment (Fig. 2A). To test how these proteins could interact within the bacterial envelope, we fractionated membrane vesicles from *D. dadantii* wild-type cells on a sucrose gradient (Fig. 3). No full-length OutD but an abundant OutD cross-reacting band of ~35 kDa was detected in the OM fractions (Fig. 3A), suggesting OutD degradation during the 60-h centrifugation (probably by the *D. dadantii* metalloproteases [65], since EDTA could not be used in the course of membrane separation). OutB colocalized with both membranes, in contrast to the bona fide IM protein TolA and the OM components OmpA porin and lipopolysaccharide (LPS). This suggests that a fraction of OutB is attached to the OM or to an OM-associated component, for example, the secretin or the peptidoglycan. The treatment of cell extracts with lysozyme, to release the OM vesicles from the PG mesh, improved the separation of the OM components, LPS and OmpA, which moved to the higher-density fractions, but OutB remained split between the two membranes (Fig. 3B). However, when the membranes of a Δ*outD* strain were separated, the effect of the PG on OutB location became clearly visible (Fig. 3C and D). Here, and only after cell wall cleavage by lysozyme, OutB was detected uniquely in the IM fractions. These data suggest that OutB attaches to both the PG and OutD. We further examined these two possibilities.

10.1128/mbio.00253-22.1 FIG S1 (A) In *D. dadantii*, the *outB* mutation diminishes the amount of OutD. The *D. dadantii* wild-type strain and *outD*, *outB, outB*_Δ_*_cte_*, *outC*, and *outG* mutants were grown for 14 h at 30°C on pectin-containing medium. An equivalent amount of cells was loaded into each well and probed by immunoblotting with the indicated antibodies. Positions of the Out proteins and molecular mass standards are indicated. (B) Schematic of the plasmids used in this study. The indicated gene combinations were cloned into pGEM-T plasmid (Promega) such as *outC* and *outD* are expressed under the control of P*lac*. (C) Domain structure of *D. dadantii* OutB, OutC, and OutD. For OutD, the upper numbering is that of the full-length polypeptide, and the lower numbering is that of the matured, signal peptide-less protein. Download FIG S1, TIF file, 2.8 MB. Copyright © 2022 Zhang et al. 2022 Zhang et al. https://creativecommons.org/licenses/by/4.0/ This content is distributed under the terms of the Creative Commons Attribution 4.0 International license.

10.1128/mbio.00253-22.8 FIG S8 (A) Unrooted phylogeny of proteins carrying the HR GspB domain (PF16537). Phylogenetic tree topology was obtained by using a bootstrapped maximum-likelihood approach with the IQ tree web server as described in Materials and Methods and visualized with iTOL software. Bootstrap values of ≥35 are indicated below the branches. GspB proteins that represent archetypes 1, 2, 4, 5, and 7 (Fig. 2B) are indicated and underlined with different colors. All the other, nonindicated cases represent the most abundant archetype, 3. GspB proteins from alpha-, beta-, and deltaproteobacteria are indicated; the others are from gammaproteobacteria. Note that GspA-GspB fusions (archetypes 4 and 5) appear in different groups. (B) Sequence logo of HR GspB domain sequences. Graphical representation of multiple sequence alignment of HR GspB domains (117 sequences) used to generate the phylogenetic tree in panel A. The height of symbols indicates the relative frequency of each amino acid at that position. The sequence logo was generated with the weblogo.berkeley.edu server. Download FIG S8, TIF file, 1.9 MB. Copyright © 2022 Zhang et al. 2022 Zhang et al. https://creativecommons.org/licenses/by/4.0/ This content is distributed under the terms of the Creative Commons Attribution 4.0 International license.

10.1128/mbio.00253-22.10 TABLE S2 Bacterial strains, plasmids, and primers used in this study. Download Table S2, DOCX file, 0.02 MB. Copyright © 2022 Zhang et al. 2022 Zhang et al. https://creativecommons.org/licenses/by/4.0/ This content is distributed under the terms of the Creative Commons Attribution 4.0 International license.

### OutB suppresses PspA induction caused by mislocated secretin.

Inefficient targeting of secretins to the OM results in their spontaneous insertion into the IM that causes ion leakage from the cytoplasm. This provokes induction of a phage shock protein (Psp) stress response system that acts to restore membrane integrity (37, 66–70). Consequently, an increased level of PspA is an indicator of inefficient targeting of secretins. In *D. dadantii* and E. coli, PspA is induced in the absence of the pilotin OutS/GspS, which promotes OutD/GspD targeting to the OM (40, 68, 69).

**FIG 4:**
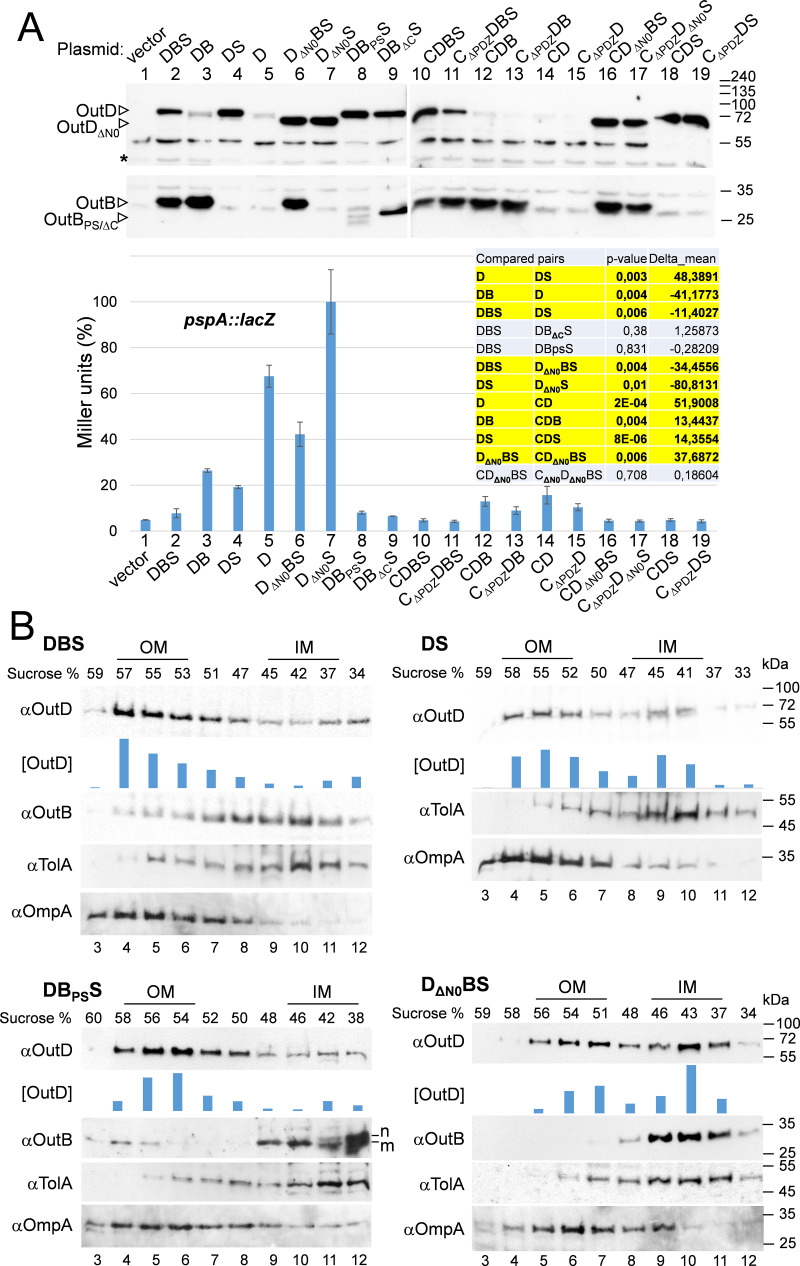
OutB suppresses the PspA response caused by OutD and assists OutD targeting in the OM. (A) E. coli MC3 carrying pREP4 (*lacI*^q^) and pGEM-T with the indicated combinations of *outC*, *outB*, *outD*, and *outS* (C, D, B, and S, respectively) were grown at 28°C for 5 h, induced with 1 mM IPTG, and grown for an additional 5 h. The amounts of OutD and OutB were estimated by immunoblotting, and the PspA level was evaluated by β-galactosidase activity measurement (*pspA*::*lacZ*). The β-galactosidase values are means from three cultures, one of which was also probed by immunoblotting shown here. *P* values were obtained with the Welch *t* test. The asterisk indicates a nonspecific cross-reacting protein, used as a loading control. (B) E. coli MC3 cells grown as for panel A were broken with a French pressure cell, and membranes were separated on a sucrose gradient and analyzed by immunoblotting with the indicated antibodies. The positions of the IM and OM are indicated according to the location of TolA and OmpA, respectively. The quantity of OutD in the fractions ([OutD]) was estimated with Image Lab software (Bio-Rad) and is shown with histograms. The bar heights are in arbitrary units and show the relative amount of OutD in each fraction, according to the pixel intensity and area of each protein band. Nonmatured and matured versions of OutB_PS_ are indicated with “n” and “m.”

To examine if OutB could also assist the OutD targeting and affect the PspA response, *outD* was expressed in E. coli either alone or with *outB* and/or *outS* from constructs designated D, DB, DBS, and DS (Fig. S1B). In this cell context, OutD remains stable enough during membrane separation in sucrose gradient centrifugation (Fig. 4). As expected, OutS caused a significant increase of the OutD level and reduced that of PspA (Fig. 4A, compare D [lane 5] with DS [lane 4]). In contrast, OutB did not consistently affect the amount of OutD but notably reduced the PspA response, and in an OutS-independent manner (Fig. 4A, compare DB [lane 3] with D [lane 5] and DBS [lane 2] with DS [lane 4]). These data suggest that OutB could improve OutD targeting to the OM. Consistent with this possibility, sucrose gradient analysis of E. coli cells producing these protein combinations showed a lower proportion of OutD in the IM fractions of the DBS sample than the DS sample (Fig. 4B). Therefore, OutB facilitates transport of OutD to the OM.

### The periplasmic OutB region decreases the PspA response caused by OutD mislocation.

OutB is composed of a short cytoplasmic region, a transmembrane helix followed in the periplasm by a linker region of variable length, and a conserved periplasmic domain, ending with a 28-residue C-terminal extension (CTE) (Fig. 2A and Fig. S1C). We examined which part of OutB is critical for reduction of the PspA response. When the CTE was deleted, the resulting protein, OutB_ΔCTE_, reduced the PspA response to a level similar to that observed with the full-length OutB, indicating that this part of OutB is not essential in this context (Fig. 4A, compare DBS [lane 2] and DB_ΔC_S [lane 9]). When the transmembrane segment of OutB was replaced with the cleavable N-terminal signal peptide, the resulting OutB_PS_ reduced the PspA response as efficiently as OutB did (Fig. 4A, compare DBS [lane 2] and DB_PS_S [lane 8]). In the sucrose gradient of DB_PS_S, OutD comigrated mainly with the OM fractions similarly to that in DBS (Fig. 4B). Notably, a small proportion of the matured, signal peptideless OutB_PS_ comigrated with the high-density OM, indicating that the periplasmic, membrane segment-free OutB_PS_ binds OutD strongly enough for them to move together through the gradient.

### OutD N0 domain is required for secretin targeting and gating the secretin channel.

In the reverse experiments, we examined which part of OutD is involved in the chaperoning by OutB. Expression of OutD_ΔN0_ lacking the N0 domain, missing 91 residues of mature OutD, from S6 to I96 (Fig. S1B), elicited a high PspA response even in the presence of OutB (Fig. 4A). Membrane separation shows that in contrast to the wild-type OutD, more than one-half of OutD_ΔN0_ comigrated with the IM fractions (Fig. 4B, compare D_ΔN0_BS to DBS). In addition, in the D_ΔN0_BS gradient, OutB was strictly confined to a few IM fractions, while in DBS (OutD_WT_), a significant proportion of OutB comigrated with the intermediate and the OM fractions. These data show that in the absence of the N0 domain, OutB is no longer able to bind OutD and assist its correct targeting.

### OutB HR domain interacts with the OutD N0 domain.

A possible direct interaction between OutB and OutD was first investigated with a glutathione *S*-transferase (GST) pulldown assay. The GST-tagged C-terminal portion of OutB, consisting of the HR and CTE (residues P112 to K220), very efficiently bound the OutD derivatives comprising the N0, N1, and N2 domains and a lone N0 domain but not to the N1-N2 derivative (Fig. S2A). Deletion of the 28-residue CTE did not affect the interaction, narrowing the interacting domains down to the HR domain of OutB (residues P112 to G192) and N0 domain of OutD (residues A1 to S85) (Fig. S2A).

10.1128/mbio.00253-22.2 FIG S2 *In vitro* experiments map the OutB-OutD interaction to OutB HR and OutD N0 domains. (A) GST pulldown with OutB HR and OutD N0 domains. The C-terminal portion of OutB (HR-CTE or HR alone) was fused to GST and coexpressed in E. coli BL21(DE3) together with the N-terminal domains of OutD (indicated at the top). The whole-cell extracts (W) were next purified on glutathione agarose (EL) and analyzed by SDS-PAGE. The protein domains and molecular mass standards are indicated. Asterisks show the positions of GST-HR-CTE (black contour) and GST-HR (white contour). (B and C) ^1^H-^15^N HSQC spectra measured with ^15^N-labeled OutB HR domain (residues P112-K220), either alone (blue spectra) or with unlabeled OutD N0-N1-N2 (residues A1 to V258) (B) or with unlabeled OutD N0 (residues A1 to S85) (red spectra) (C). The concentration of each protein was 200 μM. (D) The spectra from panel B (HR + N0-N1-N2) and from panel C (HR + N0) (red and blue, respectively) were superimposed. Of note, the peak shifts generated by N0-N1-N2 and N0 are well superimposed, indicating that only the OutD N0 domain interacts with OutB HR. Download FIG S2, TIF file, 2.6 MB. Copyright © 2022 Zhang et al. 2022 Zhang et al. https://creativecommons.org/licenses/by/4.0/ This content is distributed under the terms of the Creative Commons Attribution 4.0 International license.

### NMR characterization of the OutB HR/OutD N0 complex.

Nuclear magnetic resonance (NMR) spectroscopy was further used to explore the interaction of the OutB HR and OutD N0 domains. Initial heteronuclear single quantum coherence (HSQC) experiments using the ^15^N-labeled HR domain of OutB (residues 112 to 220) and two OutD derivatives, OutD-N0 (residues 1 to 85) and OutD-N0,N1,N2 (residues 1 to 258), showed that both OutD derivatives gave the same pattern of peak shifts, confirming that it is the N0 domain of the secretin that interacts with the HR domain of OutB (Fig. S2B to S2D). Partial assignment of the ^1^H-^15^N HSQC spectra for the OutB HR domain showed that several residues from different zones on the surface of the HR domain are affected by the interaction with the OutD N0 domain (Fig. S3A). In reciprocal experiments, residues of the OutD N0 domain involved in the interaction surface could not be readily identified from peak shifts, because when the OutB HR domain was titrated into the ^15^N-labeled N0 domain, there were widespread chemical shift changes (Fig. S3B). Such peak shifts are occasionally seen when the electron distribution is perturbed on forming a complex. Determination of the interdomain interface in the OutB HR/OutD N0 complex by NMR experiments was therefore not straightforward.

10.1128/mbio.00253-22.3 FIG S3 NMR spectroscopy analysis of the OutB HR/OutD N0 complex. (A) ^1^H-^15^N HSQC spectra of the ^15^N-labeled OutB HR domain (residues P112 to K220) alone (red) and with unlabeled OutD N0 (blue). (B) ^1^H-^15^N HSQC spectra of the ^15^N-labeled OutD N0 domain (residues A1 to S85) alone (blue) and with unlabeled OutB HR (red). Note that upon binding of HR, almost all ^1^H-^15^N signals of N0 shifted, illustrating that the chemical perturbation method is not appropriate to determine the HR/N0 interface. Download FIG S3, TIF file, 1.4 MB. Copyright © 2022 Zhang et al. 2022 Zhang et al. https://creativecommons.org/licenses/by/4.0/ This content is distributed under the terms of the Creative Commons Attribution 4.0 International license.

### Crystal structure of the periplasmic domain of OutB.

The high-resolution structures of the N0 GspDs domain have been reported for E. coli and Pseudomonas aeruginosa (31, 71). To define the molecular details of the OutB HR/OutD N0 interaction, we sought to solve the structure of the OutB HR domain. Three constructs were used in crystallization trials, OutB^112–220^, OutB^112–202^, and OutB^112–192^, of which OutB^112–202^ gave crystals used to determine the structure at a 2.05-Å resolution (Table S1). The structure of the OutB HR domain was solved using the enterotoxigenic E. coli HR GspC domain as a search model in molecular replacement (17% of sequence identity) (31). Residues 115 to 198 of OutB are clearly defined in the electron density map.

10.1128/mbio.00253-22.9 TABLE S1 Data collection and refinement statistics. Download Table S1, DOCX file, 0.01 MB. Copyright © 2022 Zhang et al. 2022 Zhang et al. https://creativecommons.org/licenses/by/4.0/ This content is distributed under the terms of the Creative Commons Attribution 4.0 International license.

**FIG 5:**
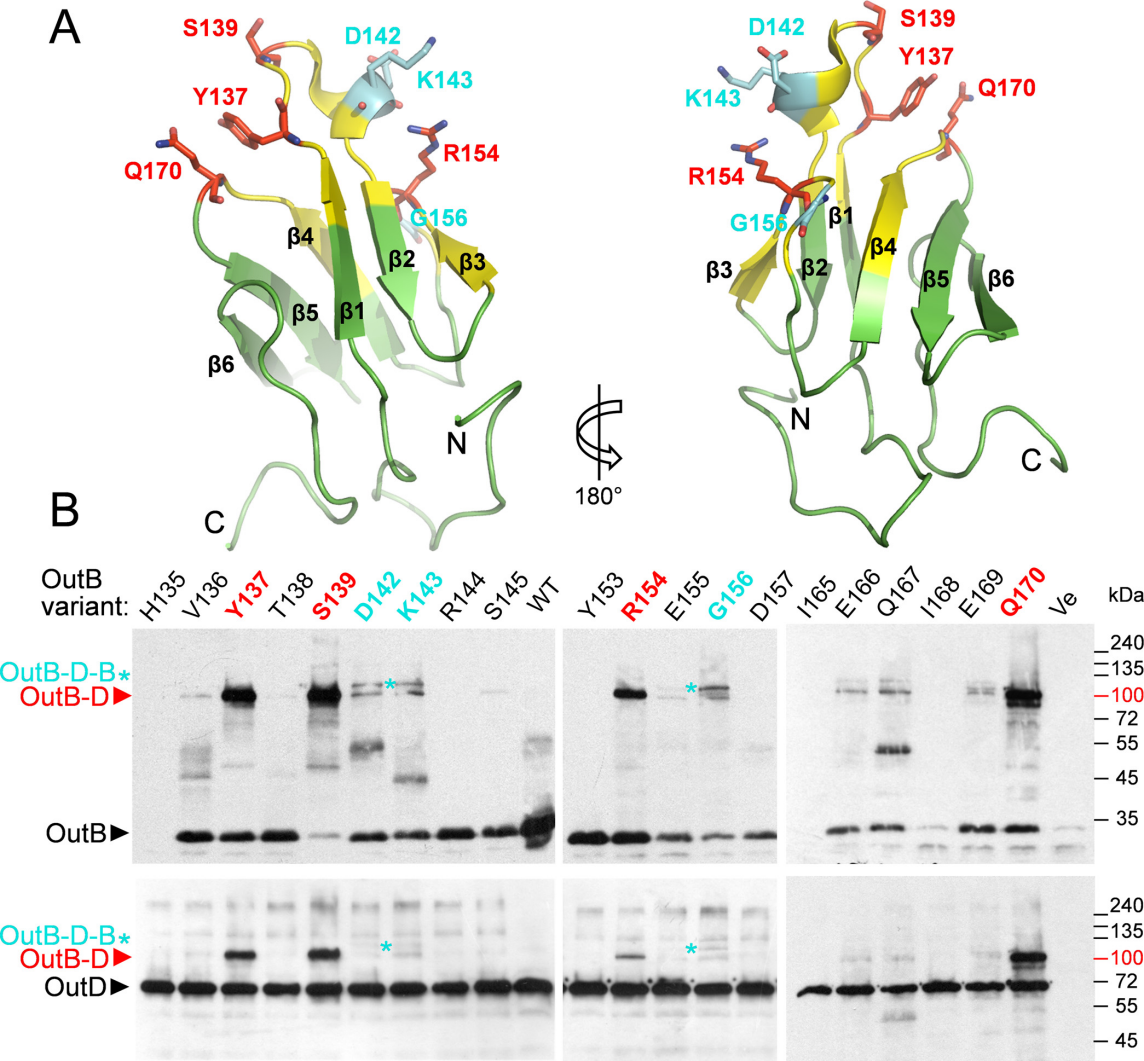
Photo-cross-linking maps OutB sites interacting with OutD. (A) The OutB HR structure is formed by two three-stranded antiparallel β-sheets coming together to form a β-sandwich. The residues generating abundant OutB-OutD complexes are in red, those generating putative OutB-D-B complexes are in cyan, and other residues probed by photo-cross-linking are in yellow. (B) *In vivo* site-specific photo-cross-linking. OutB *p*BPA substitutions (indicated at the top) were expressed from DBS plasmid in E. coli MG1655/pREP4/pSupBPA. Cells were irradiated by UV (365 nm) for 3 min and analyzed by immunoblotting with anti-OutB and -OutD antibodies (top and bottom, respectively). Only results for UV-irradiated cell extracts are shown. An equivalent amount of cells was loaded into each well. OutB, OutD, and their complexes are indicated by arrows and asterisks.

The HR domain of GspB consists of two three-stranded antiparallel β-sheets; the β-strands sequentially form the up-down-up β-sheets, so the first sheet comprises strands 1, 2, and 3, and the second, 4, 5, and 6 (Fig. 5A). The two sheets are at approximately 70° to each other, so that the structure forms a β-sandwich with a hydrophobic core. The 3_10_ helix in the loop between β1 and β2 contains a highly conserved R144, which forms a salt bridge to E155, also highly conserved (Fig. 2A and Fig. S4A). Perhaps the most striking feature of the structure is the quantity of irregular polypeptide, between the short β-strands, especially but not limited to the β3/β4 loop, and at the amino and carboxy ends of the β-sandwich (Fig. 5A). The DSSP algorithm (72) reveals that 58% of the residues do not fall into a recognized secondary-structure category.

10.1128/mbio.00253-22.4 FIG S4 The conserved periplasmic domain of OutB adopts the same topology as the HR domains of GspC T2SS and PilP T4P. (A) The crystal structure of the OutB HR domain (residues 112 to 202, PDB code 4WFW) is formed by two three-stranded antiparallel β-sheets located at approximately 70° to each other that form a β-sandwich. The loop between strands β1 and β2 forms a 3_10_ helix that is stabilized by a salt bridge between the highly conserved residues Arg144 and Glu155. (B) Structural homologues of the *D. dadantii* OutB HR: E. coli ETEC GspC HR (PDB code 3OSS), *D. dadantii* OutC HR (PDB code 2LNV), and N. meningitidis PilP (PDB code 2IVW). The cartoons are colored from the N-terminal end (blue) to the C-terminal end (red) of the polypeptide chain. (C) Structure-based alignment of the HR domains shown in panels A and B was performed with the ESPript server (107). The residue numbering is that for *D. dadantii* OutB. The secondary structure elements are shown for the aligned HR structures. The groups of identical and similar residues are shown with red highlighting and red font, respectively. Download FIG S4, TIF file, 2.0 MB. Copyright © 2022 Zhang et al. 2022 Zhang et al. https://creativecommons.org/licenses/by/4.0/ This content is distributed under the terms of the Creative Commons Attribution 4.0 International license.

A search for structural homologs using the DALI webserver (73) returned the HR GspC domain from enterotoxigenic E. coli used as the molecular replacement search model as the most similar structure (PDB entry 3OSS; Z-score, 7.8; root mean square difference [RMSD], 1.7 Å for 58 equivalent CA atoms) (31). The other related architectures are the pilot protein PilP from the T4P systems of Neisseria meningitidis (PDB entry 2IVW) and P. aeruginosa (PDB entry 2LC4) and the *D. dadantii* OutC HR (PDB entry 2LNV) (74–76) (Fig. S4B).

### *In vivo* mapping of the OutB site interacting with OutD.

To characterize the architecture of the OutB-OutD complex *in vivo*, site-specific photo-cross-linking was employed. In this approach, a photoreactive amino acid, *para*-benzoyl-phenylalanine (*p*BPA), is incorporated *in vivo* in place of the residue of interest (77, 78). Upon a short UV exposure of cells, *p*BPA can cross-link to any of the carbon-hydrogen bonds within a distance of 3 Å. In this way, we introduced *p*BPA in place of 24 residues of the OutB HR domain and assessed their cross-linked patterns in *D. dadantii* and E. coli cells. An abundant adduct of 110 kDa, cross-reacting with OutB and OutD antibodies and compatible with an OutB-OutD complex, was generated by OutB carrying *p*BPA in place of S139, Y137, Q170, and R154 (in descending order of abundance) (Fig. 5B and Fig. S5A). Remarkably, OutB_S139*p*BPA generated a near-quantitative cross-linking to OutD, indicating an important OutB-OutD contact. When OutDΔN0 lacking the N0 domain was used in place of full-length OutD, no OutB-D complex was generated, indicating that OutB HR interacts directly with OutD N0 (Fig. S5C).

10.1128/mbio.00253-22.5 FIG S5 (A) *In vivo* site-specific photo-cross-linking maps OutB HR residues interacting with OutD in *D. dadantii*. OutB *p*BPA substitutions (indicated at the top) were expressed from a DBS plasmid in *D. dadantii* Δ*outD* A3558/pREP4/pSupBPA. Cells were irradiated by UV (365 nm) for 3 min and analyzed by immunoblotting with anti-OutB and -OutD antibodies (top and bottom, respectively). (B) OutB HR structure. The residues generating abundant OutB-D complexes are in red, and other residues probed by photo-cross-linking are in yellow. (C) Photo-cross-linking shows that OutB HR interacts directly with OutD N0. OutB_R154*p*BPA was coexpressed together with OutS and with or without OutD or with OutDΔN0 and OutC, as for panel A, and analyzed by immunoblotting with anti-OutB and -OutD antibodies (top and bottom, respectively). The positions of OutB, OutD, OutB-D complexes, and molecular mass standards are indicated. (D) Photo-cross-linking of the C-terminal extension of OutB. OutB *p*BPA substitutions (indicated at the top) were expressed from a DBS plasmid as for panel A and analyzed by immunoblotting with anti-OutB antibodies. Two representative samples, T214 and K219, were then additionally treated with the lysozyme (Fig. 8B). Download FIG S5, TIF file, 1.5 MB. Copyright © 2022 Zhang et al. 2022 Zhang et al. https://creativecommons.org/licenses/by/4.0/ This content is distributed under the terms of the Creative Commons Attribution 4.0 International license.

Notably, the substitutions located at or near the β1 strand of OutB HR generated cross-linking patterns with even/odd alternation, typical for a β-strand addition (79). For instance, an OutB-D complex was generated with *p*BPA substitutions of Y137 and S139 but not with V136, T138, or A140 (Fig. 5B and Fig. S5A). These data suggest that the β1 strand of OutB HR interacts with a β-strand from OutD N0. Since Q170 (β4-β5 turn) is spatially proximal to Y137 and S139 (β1 strand), these three residues seem to constitute the main OutD-interacting zone of OutB. Another residue generating an abundant OutB-OutD complex, R154, is located rather far from this patch of highly reactive residues (Fig. 5A) suggesting formation of an extended HR/N0 interdomain interface or even two interfaces. Interestingly, some OutB_*p*BPA variants, e.g., the D142, K143, and G156 variants, generated two OutB and OutD cross-reactive adducts, one OutB-OutD and another one, of higher mass, compatible with the OutB-OutD-OutB complex (Fig. 5B). This putative ternary complex could result from a simultaneous cross-linking of one N0 domain to two HR domains.

### Mapping of the OutD site interacting with OutB.

**FIG 6:**
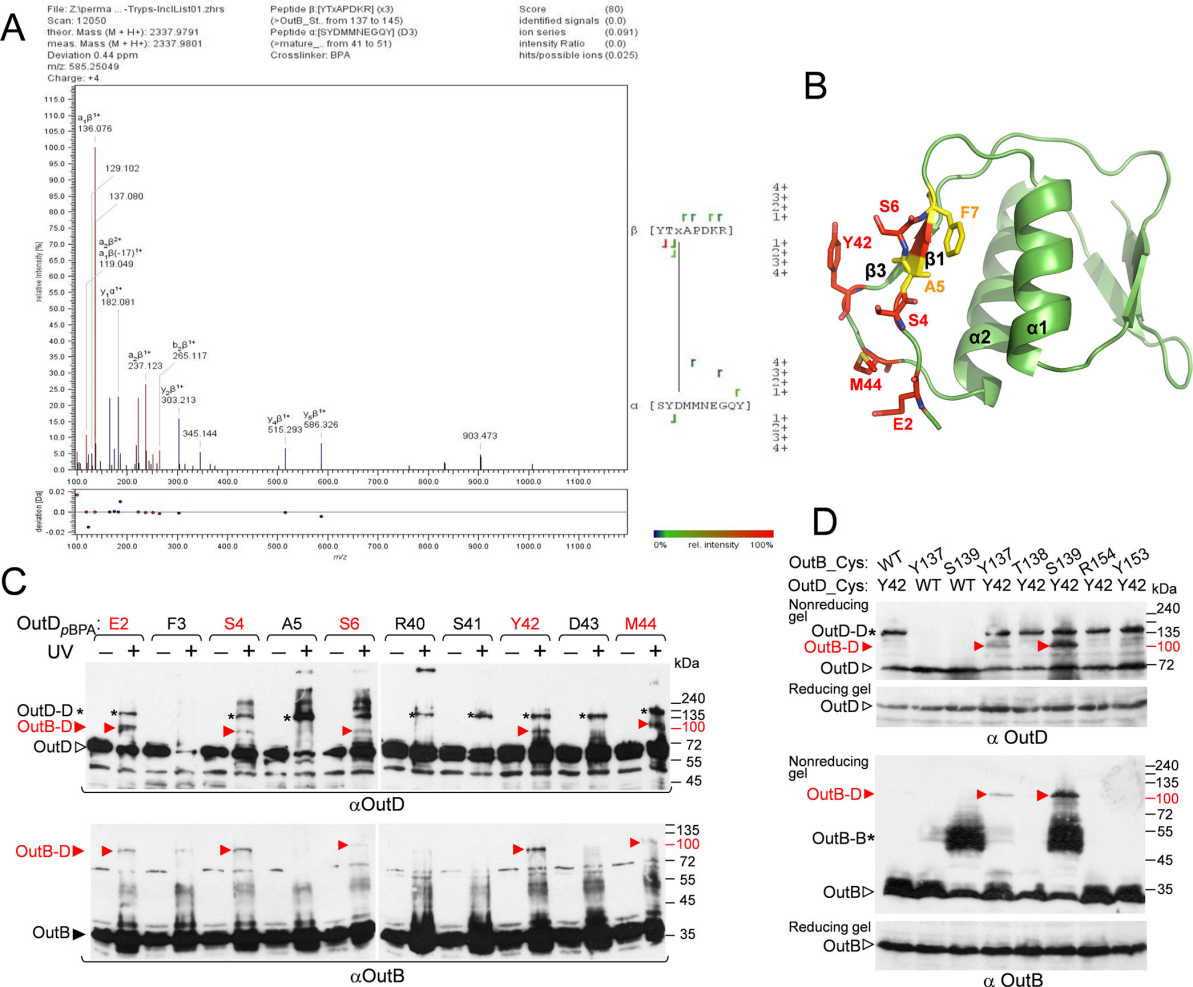
Mapping of the OutD site interacting with OutB. (A) LC-MS/MS analysis shows that OutB_S139*p*BPA interacts with the SYDMMNEGQY peptide of OutD. The OutB_S139*p*BPA-OutD complex was purified by Strep-Tactin chromatography followed by SDS-PAGE and in-gel digestion (Fig. S6). The peptides were subjected to a high-resolution LC-MS/MS analysis, and MS spectra were assigned with StavroX. Shown is a representative MS/MS spectrum with the assigned OutD (α) and OutB (β) peptides and cross-linking site, where “x” indicates the *p*BPA residue. The StavroX validation score of 80 is above the cutoff of 48. (B) OutD N0 residues probed by photo-cross-linking; those generating OutB-D and OutD-D complexes are in red and yellow, respectively. (C) *In vivo* photo-cross-linking maps the OutD residues interacting with OutB. OutD_*p*BPA substitutions (indicated at the top) were expressed from a DBS plasmid in E. coli MG1655/pREP4/pSupBPA. Cells were irradiated by UV and analyzed by immunoblotting with OutB and OutD antibodies. (D) Disulfide bonding analysis maps an OutB-OutD interacting site. E. coli MG1655/pREP4 carrying a DBS plasmid with the indicated cysteine substitutions in OutD and/or OutB were grown aerobically to allow formation of disulfide bonds in the periplasm. The remaining free thiol groups were then blocked with iodoacetamide, and the extent of disulfide bonding was assessed in a nonreducing gel, followed by immunoblotting with OutD and OutB antibodies. The same samples were analyzed in a reducing gel with 2-mercaptoethanol to estimate the quantities of OutB and OutD. An equivalent amount of cells was loaded into each well. OutB, OutD, and their complexes are indicated by arrows and asterisks.

To identify the OutD residues interacting with OutB HR, photo-cross-linked complexes generated by OutB_*p*BPA variants S139, R154, and Q170 were purified by Strep-Tactin chromatography and separated by SDS-PAGE, followed by in-gel digestion with chymotrypsin and trypsin. The peptides were then analyzed in a high-resolution liquid chromatography-tandem mass spectrometry (LC-MS/MS) Q Exactive HF mass spectrometer (Thermo Fisher Scientific) (Fig. 6A and Fig. S6). LC-MS/MS data were subjected to analysis with StavroX software tools (80), which allowed an accurate identification of OutB_S139*p*BPA-OutD cross-linked peptides. Specifically, at a 1% false discovery rate (FDR), several best spectra with a score above the score cutoff of 48, corresponding to the OutD peptide S^41^YDMMNEGQY^50^, cross-linked to the OutB peptide YTS^139^*_p_*_BPA_APDKR^144^ (Fig. 6A and Fig. S6D). The OutD residue, being cross-linked to *p*BPA, could be assigned from S^41^ to M^45^ with prevalence of D^43^ and M^44^ (Fig. S6D).

10.1128/mbio.00253-22.6 FIG S6 Mass spectrum analysis of OutB_*p*BPA-OutD complexes. (A) E. coli cells coexpressing indicated OutB_*p*BPA variants with OutD-Strep were subjected to *in vivo* photo-cross-linking and generated OutB-OutD complexes were purified by Strep-Tactin chromatography and separated by SDS-PAGE. The protein bands indicated with red triangles were excised and subjected to in-gel digestion with chymotrypsin and trypsin and analyzed by high-resolution LC-MS/MS. (B) OutD sequence coverage by the MS/MS analysis of the OutB-OutD complexes generated by OutB_Q170*p*BPA and OutB_R154*p*BPA variants. (C) The peptides that were not assessed by MS/MS analysis for panel B are highlighted with different colors on the N0 OutD sequence and structure. (D) Ten best unique cross-links identified between OutB_S139*p*BPA and OutD_strep using StavroX analysis software. Download FIG S6, TIF file, 1.7 MB. Copyright © 2022 Zhang et al. 2022 Zhang et al. https://creativecommons.org/licenses/by/4.0/ This content is distributed under the terms of the Creative Commons Attribution 4.0 International license.

To further map the OutD binding site, in reverse photo-cross-linking experiments, *p*BPA substitutions were introduced along the β1 and β3 strands of OutD N0, in place of the residues detected by the MS analysis or spatially close to them (Fig. 6B and C). An obvious OutD-OutB complex reactive with both OutD and OutB antibodies was detected with the OutD_*p*BPA substitutions of E2, S4, S6 (β1), Y42, and M44 (β3) (Fig. 6C). In the OutD N0 structure (Fig. 6B), these residues are located close to each other and hence could interact with the same or proximal sites of the OutB HR. Significantly, Y42 and M44 support the OutD cross-linking site identified by the MS analysis (Fig. 6A).

The validity of the identified HR-N0 interaction site was further assessed with an *in vivo* disulfide-bonding assay. Several residues of OutB HR that generated a *p*BPA-induced complex were substituted with cysteine and coexpressed with either the wild-type OutD or OutD_Y42C, since the OutD_M44C variant was barely detectable. Formation of disulfide bonds between spatially proximal cysteine residues were next assessed during bacterial growth. Among the tested combinations, OutB_S139C/OutD_Y42C and OutB_Y137C/OutD_Y42C pairs generated an abundant OutB-OutD complex, supporting the proximity of these residues in the functional T2SS (Fig. 6D). Therefore, in spite of the different hydrophobicity of the substituted residues, *p*BPA versus cysteine, and the length of the respective cross-linking, 3 Å versus 7 Å, both *in vivo* approaches confirm the MS data and clearly map one OutB-OutD-interacting site to S139 of OutB HR and to a short zone around Y42 and M44 of OutD N0.

### Model of OutD N0-OutB HR interaction.

**FIG 7:**
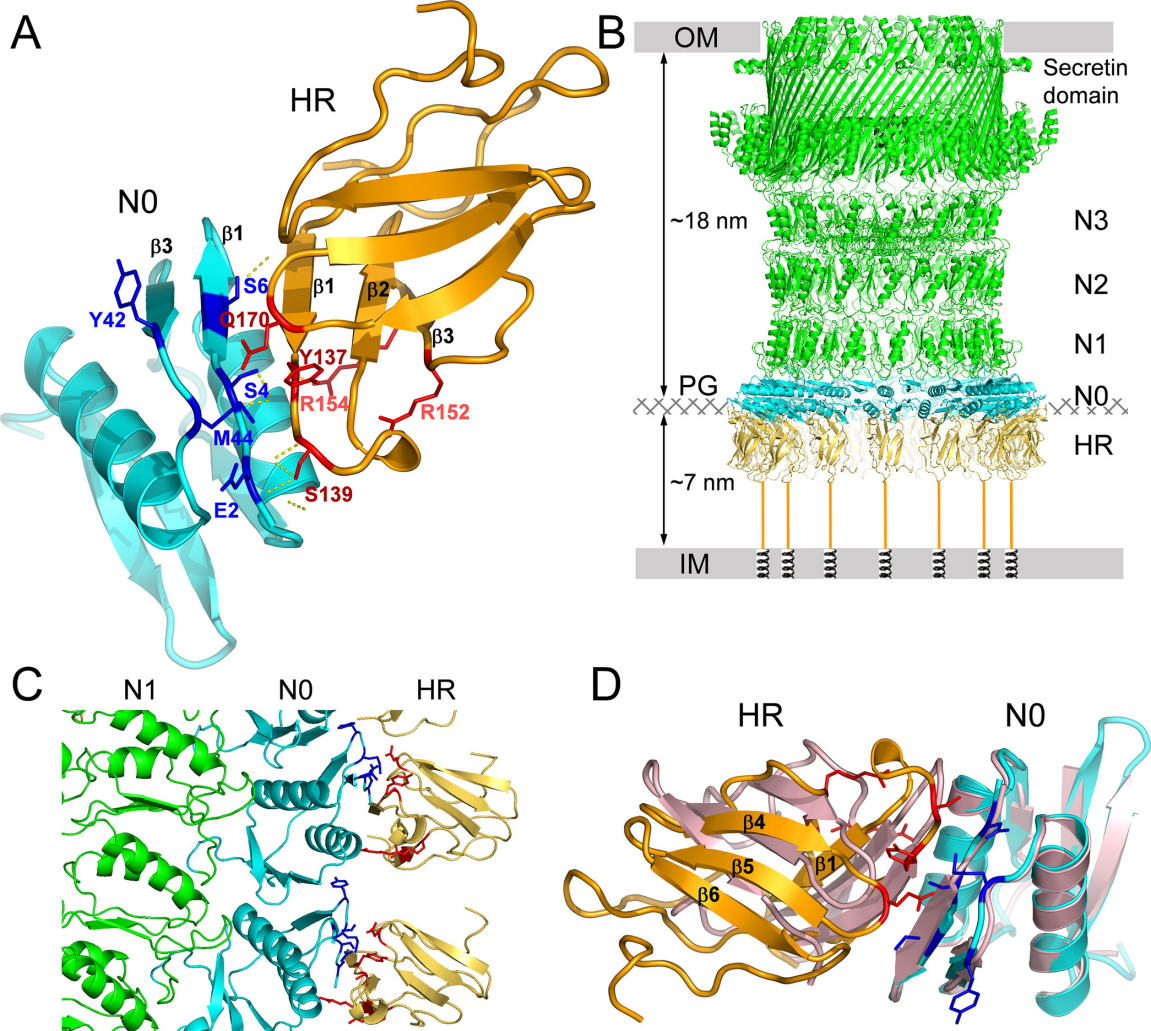
Model of the OutB HR/OutD N0 complex of *D. dadantii*. (A) OutB HR/OutD N0 interdomain interface modeled according to the *in vivo* cross-linking data. (B) Model of the 15-meric secretin channel in complex with OutB HR domains, assuming that all N0 domains are occupied, placed in the context of *D. dadantii* cell envelope. The peptidoglycan layer (PG) is positioned with respect to the outer membrane (OM) and inner membrane (IM) according to reference 1. The length of the periplasmic portion of the secretin channel (from OM to N0 domain) was estimated as described in references 21, 23, and 27 and was roughly equal to the distance from OM to PG. (C) Close-up view of the interacting regions between OutB HR and OutD N0; two adjacent N0/HR complexes are shown. OutB HR domains are in gold, OutD is in green, and N0 domains are in cyan. (D) Superimposition of the GspC HR/GspD N0-N1 complex (PDB 3OSS), in pink, with the OutB HR/OutD N0 complex in gold and cyan, respectively. The OutB HR and OutD N0 residues involved in the interdomain interface are in red and blue, respectively. The superimposition was made by using only the N0 domains as the templates.

Based on the data from *in vivo* cross-linking experiments, we generated an OutB HR/OutD N0 model by employing the HDOCK server (81). In this docking modeling, the proximity of the residues Y137, S139, R152, R154, and Q170 of OutB HR and E2, S4, S6, Y42, and M44 of OutD N0 was given as a preferable constraint for interacting residues. Among several generated OutB HR/OutD N0 models, one distinguished by an excellent docking energy of −149.45 and several convincing structural features was retained (Fig. 7A). First, in this model, all the interacting residues of OutB HR and OutD N0, listed above, are placed in close proximity. Second, a striking aspect of this model is the complementation of several β-strands between the OutB HR and OutD N0 domains. Specifically, the β1 strand of OutB HR defined by residues S133 to Y137 determines an antiparallel β-sheet with the β1 strand of OutD N0 containing residues S4 to K8; this leads to a complementary five-stranded anti-parallel β-sheet implicating the two distinct domains (Fig. 7A). Such a mixed HR/N0 β-sheet is consistent with the even/odd alternation of photo-cross-linking patterns observed with the residues of the β1 strand of the OutB HR and the β1 strand of OutD N0 (Fig. 5B and 6C). It seems plausible that formation of this mixed β-sheet together with a rather large HR/N0 interdomain interface could explain the high affinity of the OutB HR/OutD N0 interaction observed both *in vivo* and *in vitro* (Fig. 5B; Fig. S2 and S5).

The *D. dadantii* OutD is highly similar (66% sequence identity) to the secretin PulD of K. pneumoniae whose pentadecameric structure was recently solved by cryo-EM (27). The N0 domains are notoriously flexible but have been imaged in this high-resolution structure as a tightly packed ring stabilized in the presence of PulC (PDB 6HCG). Based on the PulD structure, we generated the full-length *D. dadantii* secretin model, to validate its ability to bind OutB in such a multimeric conformation. The generated model confirms that OutB is indeed able to bind to the 15-meric *D. dadantii* secretin with no clashes (Fig. 7B and C). The steric hindrance of the HR/N0 interface area allows the binding of one copy of OutB to one OutD subunit. Our protein/protein docking model between OutD and OutB HR shows that it can be repeated 15 times to form a complete ring with a relative stoichiometry of the OutB-OutD complex of 1:1. In this arrangement, OutB HR domains are located radially inward from the N0 ring without any lateral steric contact clashes with neighboring HR domains, forming a cap to the entry of the pentadecameric secretin channel.

### Search for possible competition between the HR domains of OutB and OutC.

The HR domain of GspC/OutC components has also been reported to interact with the N0 domain of secretin (31, 32). Interestingly, the OutB HR/OutD N0 complex, which we modeled based on *in vivo* cross-linking data (Fig. 7A), is similar to the crystal structure of GspC HR/GspD N0-N1 complex from E. coli (PDB entry 3OSS) (31). Particularly, when the N0 domains in these two structures were superimposed, the corresponding HR domains are also well superposed, so that equivalent secondary structure elements are placed in the two HR/N0 interdomain interfaces (Fig. 7D). It therefore seems plausible that in the secretion system, OutB and OutC could also establish such equivalent arrangements, competing for the secretin.

To test this hypothesis, NMR HSQC titration experiments were performed by mixing the OutB HR, OutC HR, and OutD N0 domains (Fig. S7A to C). Under these conditions, OutC HR did not compete with OutB HR for OutD N0, indicating that *in vitro*, the OutB HR binds to the OutD N0 domain with higher affinity than OutC HR. To examine further the OutC-OutD interactions *in vivo*, photo-cross-linking (photo-CL) was used. *p*BPA substitutions were introduced in place of several residues of the OutC HR domain, equivalent to those located in the E. coli GspC HR/GspD N0 crystal interface (31), namely, G99, M101, Q119, F120, and S121 (β1 and β3 strands of OutC HR, respectively) (Fig. S1C and S7D). In comparison with the positive control carrying the OutB_R154*p*BPA substitution, no or small amounts of high-molecular-mass species (putative OutC-OutD complexes) were detected with these OutC_*p*BPA variants with anti-OutC antibodies. The most abundant adducts were generated by OutC_G99*p*BPA and OutC_M101*p*BPA substitutions. However, no equivalent species were detected with anti-OutD antibodies (Fig. S7D, compare anti-OutC and anti-OutD panels). Furthermore, using the OutD_ΔN0_ variant instead of the full-length OutD did not alter the patterns and size of the generated species, indicating that these adducts are not OutC-OutD complexes (Fig. S7D). In addition, the presence of OutB did not apparently affect the efficiency of these photo-cross-links (Fig. S7D, compare CDS and CDBS patterns). These data suggest that the assessed residues of OutC and OutD are not close enough (≤3 Å) to be cross-linked or that the dynamics of these interactions is not compatible with photo-CL. In spite of the absence of obvious OutC-OutD complexes in photo-CL, coexpression of OutC together with OutB_*p*BPA variants caused some diminution in the quantity of the OutB-OutD complex (Fig. S7E, compare DBS and CDBS patterns of OutB_R154*p*BPA). However, such an effect was visible only in *D. dadantii* and not in E. coli, suggesting a partial spatial hindrance between OutC and OutB within the T2SS.

10.1128/mbio.00253-22.7 FIG S7 Competition between OutB HR and OutC HR domains. (A to C) The OutC HR domain does not compete efficiently with The OutB HR for OutD N0 domain *in vitro*. Chemical shifts measured with ^15^N-labeled OutB HR domain (residues P112 to K220) when unlabeled OutD N0 domain (residues A1 to S85) and OutC HR domain (residues A78 to S158) were added. The concentrations of proteins were as follow: ^15^N OutB HR, 40 μM; OutD N0, 60 μM and OutC HR, 120, 240, and 360 μM (A, B, and C, respectively). No significant signal shifts were detected with increased concentrations of OutC HR. (D) Photo-cross-linking analysis of OutC-OutD interactions. The indicated OutC*p*BPA variants were coexpressed with OutD or OutD_ΔN0_ and with or without OutB in *D. dadantii* A3556 Δ*outC*/pREP4/pSupBPA, as indicated at the tops of the panels. Cells were irradiated by UV (365 nm) for 3 min and analyzed by immunoblotting with the antibodies against OutC and OutD. Of note is that the patterns and sizes of HMW adducts of OutC_G99*p*BPA and OutC_M101*p*BPA observed with anti-OutC do not vary depending on the presence of OutD, OutD_ΔN0_, or OutB. (E) Effect of coexpression of OutC on the OutB-OutD interaction. An OutB_R154*p*BPA variant was coexpressed with OutD and with or without OutC, in either E. coli MG1655/pREP4/pSupBPA or *D. dadantii* A5654/pREP4/pSupBPA, as indicated at the tops of the panels. Cells were irradiated by UV (365 nm) for 3 min and analyzed by immunoblotting with the antibodies against OutB, OutD, and OutC. The data for a representative biological replicate are presented. In *D. dadantii*, the amount of OutB-OutD complex was steadily lower in the presence of OutC. Download FIG S7, TIF file, 1.7 MB. Copyright © 2022 Zhang et al. 2022 Zhang et al. https://creativecommons.org/licenses/by/4.0/ This content is distributed under the terms of the Creative Commons Attribution 4.0 International license.

To further evaluate the relevance of OutC interactions with the secretin, we assessed the ability of OutC to control the *pspA* response induced by mislocated secretin. Coexpression of OutC with OutD led to a very efficient decrease of PspA level, regardless of the presence of OutB (Fig. 4A; compare DB [lane 3] with CDB [lane 12] and DS [lane 4] with CDS [lane 18]). Notably, in the presence of OutC, the OutD_ΔN0_ variant (lacking the N0 domain) caused a rather weak PspA response, in contrast to that seen with OutB (Fig. 4A, compare D_ΔN0_BS [lane 6] with CD_ΔN0_BS [lane 16]). This indicates that OutC efficiently interacts with the secretin lacking the N0 domain, hence decreasing the PspA response. To test if such interactions involve the HR or PDZ of OutC, the latter domain was deleted (Fig. S1B and C). The removal of PDZ did not cause an increase in PspA level (Fig. 4A, compare CD_ΔN0_BS [lane 16] with C_ΔPDZ_D_ΔN0_BS [lane 17]), indicating that it is the OutC HR domain that interacts with the OutD_ΔN0_ variant, most probably with the N1 and/or N2 domains.

### GspB phylogeny.

A search against the UniProtKB database by using the OutB HR domain as the template shows the occurrence of GspB homologs in several groups of gammaproteobacteria and betaproteobacteria. All these proteins carry a single transmembrane segment and a periplasmic HR domain but vary substantially in length and composition, so that a confident phylogenetic analysis could be performed only with the HR domains of these proteins (Fig. S8). Systematic inspection of *gspB* synteny and domain organization allow us to classify these proteins into seven archetypes (Fig. 2B). In most bacteria, *gspB* is colocated with *gspA.* Typically, GspA consists of a cytoplasmic AAA+ ATPase domain fused to a C-terminal transmembrane segment that is followed by a large periplasmic region with a cysteine peptidase-like and a PG-binding domain, such as in *Vibrio* and *Aeromonas* (Fig. 2B). However, in *Alteromonas* and *Pseudoalteromonas*, GspAs lack the periplasmic region and the cognate GspBs are notably longer than in the other bacteria (Fig. 2B). In addition, GspBs of *Alteromonas* possess a supplementary cytoplasmic domain of DUF3391 family. Interestingly, in these bacteria, a gene coding for a DUF3391 domain protein is present next to *gspB* (Fig. 2B), indicating a possible origin of the DUF3391 domain in GspB. In some bacteria, such as Vibrio vulnificus, GspA is fused to the periplasmic portion of GspB in a single GspAB polypeptide (82). Similar GspAB fusions of various lengths and domain contents seem to have appeared independently in several groups of gammaproteobacteria (Fig. S8A), supporting a close functional relationship between GspA and GspB. E. coli K-12 and some other closely related enterobacteria possess an archetypal multidomain GspA paired with a remarkably small GspB that has no HR domain but carries instead a variable polyampholyte sequence (Fig. 2B). Finally, *Dickeya*, *Pectobacterium*, Klebsiella, and a few other close bacteria lack any *gspA* homolog, and *gspB* is colocated with a gene of the OutS/PulS pilotin family. Therefore, depending on the bacterium, GspB proteins vary substantially in length and domain composition and act in concert with either a cognate GspA or a pilotin GspS.

### OutB can be efficiently cross-linked to the PG *in vivo*.

Separation of *D. dadantii* membrane vesicles in a sucrose gradient suggested that OutB is anchored to the PG. Notably, degradation of the PG by lysozyme affects the location of OutB (Fig. 3C and D). In *Aeromonas* and *Vibrio*, GspA carries a specialized PG-binding domain (Fig. 2B) and the GspA/GspB complex is involved in the insertion of cognate secretins into or through the PG mesh (57, 83). In *Dickeya* and related bacteria that lack a GspA homolog, OutB has an additional C-terminal polyampholyte sequence (Fig. 2). Therefore, we investigated if in *D. dadantii*, the absence of GspA could be compensated for by this C-terminal extension.

**FIG 8:**
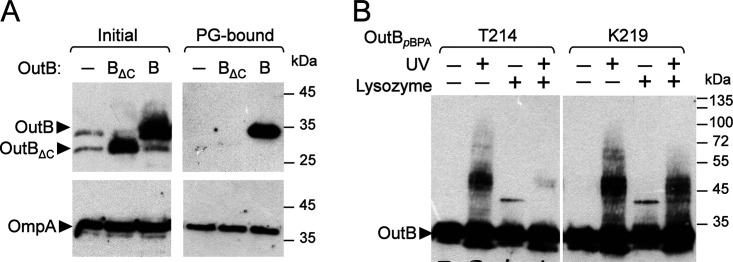
OutB interacts with peptidoglycan. (A) *In vivo* chemical cross-linking with DTSSP. *D. dadantii outB* A5719 cells carrying either an empty vector or a plasmid with *outB* or *outB*Δ*cte* (−, B, and B_ΔC_, respectively [“Initial”]) were treated with DTSSP. The PG was next extracted by an SDS boiling procedure, and PG-bound proteins were eluted with 2-mercaptoethanol and analyzed by immunoblotting with anti-OutB or anti-OmpA (“PG-bound”). Twenty-five-fold less material, in cell equivalents, was loaded in the “Initial” gel than the “PG-bound” gel. (B) *In vivo* site-specific photo-cross-linking. OutB *p*BPA substitutions (T214 and K219) were expressed in *D. dadantii outB* A5719. Cells were irradiated by UV, treated with lysozyme or left untreated, and analyzed by immunoblotting with anti-OutB.

The thiol-cleavable cross-linker 3,3′-dithio-bis(sulfosuccinimidyl)propionate (DTSSP) was used to explore OutB-peptidoglycan interaction. *D. dadantii* Δ*outB* cells harboring a plasmid with *outB*, *outB*Δ*cte*, or empty vector were treated with DTSSP, and PG was extracted by an SDS boiling procedure. The PG-bound proteins were next eluted with 2-mercaptoethanol and analyzed by immunoblotting (Fig. 8A). The PG-associated protein OmpA, used as a positive control, was equally eluted from all three PG preparations. In contrast, only OutB and not OutB_ΔCTE_ was massively eluted from the PG. Therefore, OutB was efficiently cross-linked by DTSSP to the PG via its C-terminal extension (CTE), suggesting that the OutB CTE is proximal to the PG layer and could interact with it.

To further explore the OutB-PG interaction, photo-cross-linking with *p*BPA, which has a shorter arm length than DTSSP (3 Å versus 12 Å) and a shorter time lapse (3 min versus 20 min), was employed. *p*BPA substitutions were introduced into several residues of OutB CTE, and their cross-linking patterns were assessed in *D. dadantii* (Fig. 8B and Fig. S5D). Diffuse multiband adducts of about 40 to 50 kDa over a less intense ladder-like background were observed. Treatment of cross-linked cells with lysozyme significantly decreased the amount of diffuse adduct and reduced the ladder-like background, indicating that the PG-derived sugars are part of these complexes. Taken together, these data suggest that *in vivo*, the C-terminal extension of OutB interacts with the PG. The functional relevance of the CTE was shown using the *D. dadantii outB*Δ*cte* mutant, where the reduced pectinase halos indicate diminished secretion (Fig. 1B). The effect was not as substantial as the complete *outB* deletion but was repeatable, revealing the functional importance of the C-terminal extension of OutB.

## DISCUSSION

Here, we show that OutB is essential for effective assembly and function of the *D. dadantii* T2SS and for expression of full bacterial virulence. We reveal that OutB has three functions linked to distinct regions of the protein. (i) The N-terminal transmembrane helix anchors OutB to the IM. (ii) The conserved periplasmic domain of OutB (HR) tightly binds the N0 domain of the secretin OutD and assists its targeting to the outer membrane. (iii) The C-terminal region (CTE) attaches OutB to the peptidoglycan, anchoring the entire secretion system to the bacterial cell wall.

The crystal structure reveals that the conserved periplasmic domain of OutB has the same fold as the homology regions (HR) of the T2SS GspC and the T4P PilP components (31, 74–76). In spite of low sequence conservation (10% to 17% of identical residues), OutB HR structure is highly similar to these HR domains (RMSD, 1.7 Å to 2.8 Å for 58 equivalent CA atoms). This structural similarity coincides with the fact that all these HR domains have been reported to interact with the N0 domain of cognate secretins (31, 32, 74, 84). By combining structural, *in vivo*, and modeling approaches, we unraveled the molecular details of the OutB HR/OutD N0 interaction. Specifically, site-specific photo-cross-linking followed by mass spectrometry analysis allowed an unambiguous identification of the OutB-OutD interaction site *in vivo*. The validity of this site was further confirmed with reverse photo-cross-linking and *in vivo* disulfide bonding. Consequently, the OutB HR/OutD N0 model constructed on the basis of these data reflects a functionally relevant protein contact within the functional secretion system. NMR spectroscopy was roughly consistent with the cross-linking experiments but for various technical reasons failed to give a comprehensive list of residues involved in the OutB HR/OutD N0 interface.

The N0 domains constitute the entrance of the T2SS secretin channel, which controls the recruitment of substrates and communicates with the IM components of the secretion machinery (29–32). Recent cryo-EM tomography of the L. pneumophila T2SS showed high flexibility of the N-terminal portion of the secretin channel, which could reflect different functional states and interactions with various partners (34). The occurrence of two components, OutB and OutC, carrying structurally similar HR domains that seem to interact with the same N0 domain of the cognate secretin raises the questions of the hierarchy of these interactions within the T2SS.

The fact that the OutB HR/OutD N0 interface modeled after *in vivo* photo-CL data is strikingly similar to that of the crystal GspC HR/GspD N0-N1 complex (Fig. 7D) (31) suggests that these two interactions are spatially incompatible and hence could not occur simultaneously. However, several lines of evidence indicate that *in vivo*, the HR domain of OutC adopts a more complex mode of interaction with the secretin than HR of OutB. First, the absence of any abundant OutC-OutD complex in photo-CL experiments suggests that *in vivo*, the OutC and OutD residues selected according to the crystal GspC HR/GspD N0 interface (31) are not close enough (≤3 Å), or that such an interface is highly dynamic. Second, NMR experiments show that *in vitro*, the OutB HR binds OutD N0 more tightly than the OutC HR does, with affinities in the range of 1 mM for OutC HR versus 10 μM for OutB HR (Fig. S7A to C). Third, PspA induction assays show that OutC_ΔPDZ_ efficiently rescues OutD lacking the N0 domain, thus indicating that the OutC HR domain interacts with another domain(s) of the secretin. These data support a previous disulfide bonding analysis in *D. dadantii* that showed interactions of OutC with N1 and N2 domains of the secretin (32). Therefore, it is tempting to hypothesize that the crystal GspC HR/GspD N0 interface (31) is a snapshot reflecting one of several possible arrangements of OutC and OutD in the functional T2SS. Consistent with this assumption, a previous NMR structural study revealed a somewhat different arrangement of the OutC HR/OutD N0 domains (74). It should also be noted that the presence of OutC did not affect the efficiency of OutB-OutD photo-CL in E. coli (Fig. S7D) and only slightly interfered with it in *D. dadantii* (Fig. S5C and S7D), indicating a partial spatial hindrance between OutC and OutB. It seems likely that in the T2SS, the interactions of OutB and OutC with the secretin could be temporally separated. Indeed, since OutC is involved in substrate recruitment (29, 30), OutC-OutD interactions could be prevalent during the course of secretion while OutB-OutD interactions play a role in the assembly, maintenance, and scaffolding of the secretin channel.

We present a model of the OutD N0/OutB HR interaction in the context of the 15-meric secretin channel (Fig. 7B). In this model, each OutB HR binds one OutD N0 domain in a 1:1 ratio, so that the OutB HR domains are located radially inward of the N0 ring with no lateral contacts with neighboring HR domains. The model presented here is therefore rather symmetric and constrained compared to the more open and dynamic arrangement, as it can be anticipated in the functional secretion system. Indeed, the functional oligomeric state of the secretin channel within the functional T2SS has not been ultimately determined. Recent cryo-EM studies revealed a C15 symmetry of the T2SS secretins (15, 21, 23, 28), while *in vivo* disulfide-bonding analyses are consistent with a C6 symmetry of a hexamer of dimers (32, 71). In agreement with the latter, the N-terminal portion of the P. aeruginosa secretin XcpQ, comprising the N0, N1, and N2 domains, self assembles into a hexamer of dimers, forming *in vitro* C6 symmetry ring-shaped channels (85). Hay and colleagues proposed an elegant solution to this apparent discrepancy (23), suggesting that the C15 symmetry observed in the secretin and N3 domains is followed by pseudo-6-fold symmetry for the N0, N1, and N2 domains, compatible with C12 or C6 symmetry of the assembly platform, either hexameric, as in the case of GspE, GspL, and GspM, or dodecameric, as in the case of GspC (27). The common symmetry element between C6, C12, and C15 symmetries is a 3-fold axis. Therefore, C12 and C15 complexes could have common 3-fold symmetry if every fifth N0 domain is unoccupied. However, it is not clear how such an arrangement would be enforced; thus, it is plausible that the complex overall is asymmetric unless 3-fold symmetry is imposed in the periplasm by other components.

The role that OutB plays to maintain the secretin channels is closely linked to its ability to bind peptidoglycan. Chemical cross-linking experiments with DTSSP and *p*BPA showed that the C-terminal extension of OutB interacts with the peptidoglycan. The PG is composed of glycan chains connected by short peptides that together form a mesh surrounding bacterial cell. In proteobacteria, the PG layer is located in the periplasm and is attached to the OM through a covalent linkage to Braun’s lipoprotein, Lpp (86). Several multiprotein structures and machineries span the entire cell envelope. The size of the peptidoglycan mesh (about 2 nm) is not compatible with the external dimensions of these large complexes, and specialized or housekeeping transglycosylases are recruited for the local rearrangement of the peptidoglycan layer in the course of their assembly through the cell envelope (87, 88). The attachment of these transenvelope machineries to the PG mesh is usually ensured by specialized PG-binding modules. For instance, the T4P system possesses two components carrying specialized PG-binding domains, the secretin PilQ and TsaP, showing the importance of a solid attachment to the PG (25, 52, 53). Generally, the PG-binding components represent a less conserved part of respective transenvelope systems, suggesting that anchorage to the PG evolved and diversified more recently, in accordance with the particular cell wall context (55). For instance, *in silico* analysis of the T6SSs revealed surprising richness and variability of the associated PG-binding modules (89). The authors of that study proposed the notion of more or less “evolved” PG-binding modules that have developed from a few ancestral proteins by gene fusion and the loss of some nonessential regions.

Phylogenetic analysis shows that in many bacteria, such as *Vibrio* and *Aeromonas*, GspB acts in concert with a multidomain ATPase GspA that carries a specialized PG-binding domain (Fig. 2B and Fig. S8). In this group, some “more evolved” GspA and GspB proteins are naturally fused into a single polypeptide (59). A C-terminal extension (CTE) similar to that of OutB is present only in the *Dickeya*/Klebsiella group, which lacks a GspA counterpart (Fig. 2). We showed that the OutB CTE could interact with peptidoglycan. Therefore, it is plausible that in these GspBs, the absence of the PG-binding partner GspA is compensated for by a polyampholyte C-terminal sequence that interacts with PG. The molecular mechanism of this interaction remains unclear, since the OutB CTE does not correspond to any known PG-binding domain. However, the residue combinations of OutB CTE are reminiscent of those in some sugar binding lectin domains. For instance, the bactericidal C-type lectins of RegIII family recognize the PG carbohydrate moiety via an EPN-like motif located at an extended loop region, and variations of this motif (EPN, QPD, EPS, etc.) alter its sugar binding specificity (90). It is possible that the OutB C-terminal extension could bind peptidoglycan in a similar way. Some other bacterial proteins, apparently lacking a specialized PG-binding domain, have been shown to bind the PG, e.g., the pilotin InvH from the T3SS of Salmonella enterica (91). Interestingly, in the Legionella pneumophila T2SS, which lacks any GspB or GspA homolog, the secretin LspD itself carries a SPOR-like PG-binding domain at the N terminus, prior to the N0 domain (34). Thus, the modes of attachment of T2SS secretins to the PG vary in different bacteria.

**FIG 9:**
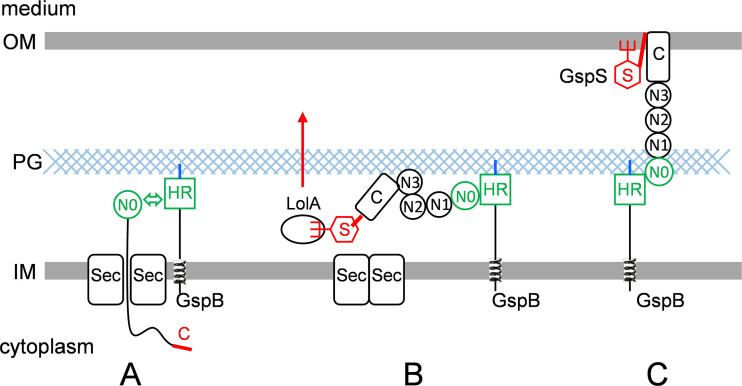
OutB and OutS have complementary functions in targeting, assembly, and scaffolding of secretin. (A) OutB acts early during OutD export by binding the N0 domain, the first part of the secretin released into the periplasm. In this way, OutB could orient the secretin and prevent its misinsertion into the IM. (B) In the later step, the pilotin OutS (red hexagon) binds the C-terminal S-domain (red segment) and, with the assistance of the Lol machinery, targets the C-terminal part of the secretin into the OM, where several secretin subunits form a channel. (C) OutB binds to and stabilizes the N-terminal entry of the secretin channel, providing scaffolding with the IM and the PG layer. For simplicity, only one subunit of each protein is shown.

In the *Dickeya*/Klebsiella group, *gspB* is colocated with the gene coding for the pilotin GspS (Fig. 2B). We propose a model wherein OutB and OutS have complementary functions in targeting, assembly, and scaffolding of the secretin (Fig. 9). OutB could act early during OutD export by binding the N0 domain, the first part of the secretin released into the periplasm. In this way, OutB could orient the secretin and prevent its misinsertion into the IM. In the later step, pilotin binding to the C-terminal S-domain, with the assistance of the Lol machinery, would target and anchor the C-terminal portion of the secretin into the OM (36), while the N-terminal entry of the secretin pore remains connected to the IM via OutB. At the same time, the C-terminal extension of OutB would attach the secretin to the PG. Therefore, GspB/OutB has a major role in the assembly of the secretin channel, providing it scaffolding with the inner membrane assembly platform and the peptidoglycan layer.

## MATERIALS AND METHODS

### Strains, plasmids, and growth conditions.

The bacterial strains and plasmids used in this study are listed in Table S2. *D. dadantii* mutant strains carrying chromosomal *outB* or *outD* mutant alleles were constructed by marker exchange-eviction mutagenesis or by homologous recombination as described elsewhere (92). The bacteria were grown in Luria-Bertani (LB) or M9 minimal medium at 28°C with shaking at 120 rpm. If necessary, glucose, glycerol, or galacturonate was added at 0.2% and antibiotics were added at the following final concentrations: ampicillin, 100 μg/mL^−1^; kanamycin, 50 μg/mL^−1^; and chloramphenicol, 25 μg/mL^−1^. DNA manipulations were carried out using standard methods. Site-directed mutagenesis was performed with the PrimeSTAR Max DNA polymerase (TaKaRa), and the primers are listed in Table S2. The sequences of mutant and amplified genes were checked (Eurofins MWG Operon).

### Protein purification.

DNA sequence encoding the conserved periplasmic domain of *D. dadantii* OutB was cloned into the pGEX-6P-3 vector (GE Healthcare) encoding a cleavable N-terminal glutathione *S*-transferase (GST) affinity tag (Table S2). Three GST-OutB constructs encoding OutB residues 112 to 220, 112 to 202, and 112 to 192 were expressed in E. coli BL21(DE3) cells (Stratagene). The bacteria were grown in Luria broth supplemented with 100 μg/mL ampicillin to an optical density at 600 nm (OD_600_) of 0.6, induced with 1 mM isopropyl-β-d-thiogalactopyranoside (IPTG), and grown for an additional 16 h at 20°C. The cells were suspended in phosphate-buffered saline (PBS) containing 0.01 M phosphate buffer, 0.0027 M KCl, and 0.137 M NaCl (pH 7.4) and lysed by sonication. Cell debris was removed by centrifugation at 35,000 × *g* for 20 min, and the supernatant from 1 L of cell culture was incubated with 2.5 mL of glutathione Sepharose beads (GE Healthcare) at 4°C for 6 h. The glutathione Sepharose beads were then collected in a PD10 column and washed with 50 mL of PBS. The GST tag was on-column cleaved by addition of 20 μL of PreScission protease to 5 mL of glutathione Sepharose in buffer containing 50 mM Tris-HCl, 150 mM NaCl, 1 mM EDTA, and 1 mM dithiothreitol (DTT) (pH 7.0) at 4°C for 6 h. N0 and N0-N1-N2 domains of OutD (residues 1 to 85 and 1 to 258 of signal peptide-less OutD, respectively) (Table S2) were produced in E. coli BL21(DE3) and purified by nickel-affinity chromatography as described previously (33). For NMR spectroscopy, uniformly ^15^N- and ^13^C-labeled proteins were produced by growing cell cultures in M9 minimal medium that contained 1 g/L [^15^N]ammonium chloride and 2 g/L d-[^13^C]glucose (Cambridge Isotope Laboratories, Inc.) as the sources of nitrogen and carbon, respectively. Prior to crystallization trials and NMR spectroscopy, the purified proteins were run on Superdex S200 size exclusion columns and concentrated with Vivaspin devices (Sartorius).

### Crystallization.

Crystallization conditions were screened using hanging-drop vapor diffusion in 96-well plates set up with a Nanodrop Mosquito liquid handling robot (TTP Labtech). Protein and precipitant drops were in the range of 200 to 400 nL. OutB (residues 112 to 202) crystals suitable for X-ray analysis were grown in a solution containing 0.2 M MgCl_2_, 0.1 M Tris, and 30% (wt/vol) polyethylene glycol (PEG) 4000, pH 8.5 (Molecular Dimensions Structure Screen 1, condition C9). Selected crystals were transferred to a solution containing mother liquor plus 5% propane-1,2-diol as a cryoprotectant and flash cooled in liquid nitrogen. Diffraction data were collected at 100 K using a Pilatus 300M detector at beamline I04 at Diamond Light Source (DLS), Oxfordshire, United Kingdom. Data were integrated and scaled by XDS through the Xia2 software package (93) available through DLS. The structure was solved by molecular replacement using Phaser from the CCP4 software package (94), using as a search model the E. coli GspC HR structure (PDB code 3OSS) detected using PHYRE2 (95). The model was refined using REFMAC v5.8 (96), and 10% of the data were excluded to be used for validation purposes.

### NMR.

NMR spectra were acquired using the shortest OutB HR domain (residues 112 to 193) at 15°C using Bruker Avance 600- and 700-MHz spectrometers equipped for ^1^H, ^15^N, ^13^C triple-resonance experiments. All spectra were processed using NMRPipe/NMRDraw (97) and analyzed using XEASY (98). HNCA, HN(CO)CA, HNCO, HNCACB, and CBCA(CO)NH experiments were used to obtain sequence-specific ^1^HN, ^15^N, ^13^Cα, ^13^Cβ, and ^13^C′ backbone assignments. The NMR samples had a typical concentration of 0.2 mM in 20 mm Tris-HCl (pH 7.0) and 10% D_2_O. NMR titration was achieved by recording a ^1^H-^15^N HSQC/^1^H-^15^N Sofast-HMQC spectrum of the labeled protein. Cross titrations were used to eliminate the dilution of the labeled protein. In cross titration, two samples were prepared; one sample was labeled protein only and the second sample had the same concentration of labeled protein and a 10 equivalents of unlabeled titration partner. NaCl (150 mM) was included in the cross-titration studies.

### Structure modeling.

A homology modeling of the *D. dadantii* OutD N0 domain (residues 1 to 78 of the matured, signal peptide-less OutD; UniProtKB entry Q01565) was conducted using the trRosetta server (99) with restraints from both deep learning and homologous templates. The confidence of the predicted model is optimal, with a TM score of 0.912 of a maximum of 1.0. Using this OutD N0 model, we then performed a protein/protein docking with the HR domain of OutB (PDB entry 4WFW). For this, the HDOCK server (81) was employed, with the contacting residues, identified by the photo-cross-linking experiments, given as preferable constraints (namely, Y137, S139, R152, R154, and Q170 of OutB HR and E2, S4, S6, Y42, and M44 of OutD N0). In addition, the docking algorithm identified PDB entry 3OSS as the template (the crystal structure of the enterotoxigenic E. coli GspC-GspD complex). This gave rise to several distinct models, one being distinguished by an excellent docking energy of −149.45 and several convincing structural features. This model was retained for subsequent steps.

A homology modeling of the full-length OutD (residues 1 to 683) was then produced using again the trRosetta server (99). A good model with a TM-score of 0.597 was produced. We then reconstructed the full-length OutD/OutB HR domain interface using the previously identified protein/protein docking pose. Finally, this OutD/OutB HR model was employed to reconstruct the *D. dadantii* secretin pentadecameric model. For this, we used the cryo-EM structure of the K. pneumoniae type II secretion system outer membrane complex (PDB entry 6HCG) as the template (27). We superimposed the OutD/OutB HR oligomer model on each of the 15 units of PulD with PyMOL v1.3 (PyMOL molecular graphics system; Schrödinger, LLC) using its cealign function, which is very robust for 3D structural alignments of proteins with little to no sequence similarity. We therefore obtained the 15-mer model of the OutD/OutB HR, and we concluded that no further minimization step was needed, as its visual inspection demonstrated that no clashes were present in the N-terminal area harboring the OutD/OutB HR interface.

### Pulldown assays.

OutB domains of interest fused to GST were coexpressed with various combinations of N0 to N3 domains of OutD from the same plasmid (Table S2) in E. coli BL21(DE3) and copurified by the batch method as described elsewhere (100). Briefly, the cells were broken in a French pressure cell in TSE buffer (50 mM Tris-HCl pH 8.0, 100 mM NaCl, 1 mM EDTA, 1 mM phenylmethylsulfonyl fluoride [PMSF]) supplemented with 0.1% (vol/vol) Triton X-100, and incubated with glutathione agarose (Macherey Nagel). Nonbound proteins were washed away six times with the same buffer, and the bound proteins were eluted with Laemmli sample buffer, separated by SDS-PAGE, and stained with Coomassie blue or probed by immunoblotting with anti-OutD antibodies.

### Site-specific *in vivo* photo-cross-linking.

*D. dadantii* or E. coli MG1655 cells, carrying pSup-BpaRS-6TRN (77), pREP4 (Qiagen), and a DBS-derived plasmid carrying an *outB_TAG* or *outD_TAG* substitution were grown in LB supplemented with ampicillin, kanamycin, and chloramphenicol at 28°C for 16 h. Cultures (1 to 1.2 mL) were then collected, washed in M9 medium, and diluted (~3-fold) to an OD_600_ of 0.5 into M9 medium containing 1 mM *p*BPA, 0.2% glycerol, 0.01% Casamino Acids, and appropriate antibiotics. To induce synthesis of pectinases and Out proteins, *D. dadantii* cultures were additionally supplemented with 0.2% galacturonate. After 1 h of growth, IPTG was added to 1 mM, and the cultures were followed for an additional 3 h or 5 h for E. coli and *D. dadantii*, respectively. Afterward, a 1.4-mL portion of cells was either chilled on ice (control) or placed in a glass petri dish and irradiated with an UV lamp (Bio-Link BLX model; 365 nm) for 3 min at a 10-cm distance. The cells were chilled on ice, collected by centrifugation, lysed in Laemmli sample buffer, and resolved on 9% SDS-PAGE. The proteins were detected by immunoblotting using antibodies raised against OutB or OutD.

### Purification of OutB-OutD complex for mass spectrometry analysis.

E. coli MG1655 cells, carrying pSup-BpaRS-6TRN (77), pREP4 (Qiagen), and a DBS-derived plasmid coexpressing an *outB_TAG* variant together with *outD_Strep*, were grown and subjected to photo-cross-linking exactly as described above. Specifically, a series of 10 tubes with 5 mL culture was grown and exposed together to UV irradiation in nine 10-cm-diameter petri dishes in the Bio-Link BLX chamber. After 3 min irradiation with UV at 365 nm, the cells were chilled on ice, collected by centrifugation, and stored at −20°C. The frozen cells from each series (50 mL culture) were resuspended in 4 mL TSE buffer supplemented with 1 mg/mL lysozyme and incubated for 15 min. The cell suspension was next supplemented with SDS to 1% and boiled for an additional 15 min. Nonsoluble cell material was removed by centrifugation at 7,000 × *g* for 10 min, and the supernatant was diluted 10-fold with TSE buffer supplemented with 1% Triton X-100 and incubated with 2 mL of Strep-Tactin XT Superflow (IBA) resin for 2 h at 15°C. The resin was next washed five times with 10 mL of the same buffer, and OutD-strep protein and complexes were eluted with TSE buffer supplemented with 1% Triton X-100 and 50 mM biotin. The eluted proteins were precipitated with 5 volumes of ethanol, resuspended in Laemmli sample buffer, and separated by 9% SDS-PAGE.

### Liquid chromatography-tandem mass spectrometry of OutB_*p*BPA-OutD complexes.

The protein bands corresponding to OutB_*p*BPA-OutD complexes were excised from a stained gel and subjected to digestion with chymotrypsin and trypsin. Specifically, the gel pieces were destained with a 50% (vol/vol) mixture of 50 mM ammonium bicarbonate in acetonitrile, reduced with 10 mM DTT (56°C, 1 h) and alkylated with 55 mM iodoacetamide (25°C, 45 min, in darkness). Proteins were first digested with chymotrypsin (Promega) (12.5 ng/μL, 25°C overnight). One-tenth of the sample was kept for injection and 9/10 was digested with trypsin (Promega) (12.5 ng/μL, 37°C, overnight). After quenching with formic acid (FA) to 5%, the two digests were analyzed with an Ultimate 3000 nano-RSLC (Thermo Fisher Scientific) coupled on line with a Q Exactive HF mass spectrometer via a nano-electrospray ionization source (Thermo Fisher Scientific).

The samples were loaded on an Acclaim PepMap100 C_18_ trap column (20 by 0.075 mm, 100-Å pore size) (Thermo Fisher Scientific) for 3 min at 5 μL/min with 2% acetonitrile, 0.05% trifluoracetic acid (TFA) in H_2_O and then separated on an Acclaim PepMap 100 C_18_ analytical column (50 by 0.075 mm, 100-Å pore size) (Thermo Fisher Scientific) with a linear gradient of solvents A (H_2_O, 0.1% FA) and B (100% acetonitrile, 0.1% FA). Settings were as follows: initial conditions of 4% to 50% of B in 60 min, then from 50% to 95% B in 2 min, hold for 10 min, and return to the initial conditions in 1 min for 14 min. The total run duration was set to 90 min at a flow rate of 300 nL/min. The oven temperature was kept at 40°C.

### Mass spectrometry data processing.

Samples were analyzed with a TOP20 Higher-energy Collisional Dissociation (HCD) method. MS data were acquired in a data-dependent strategy, selecting the fragmentation events based on the 20 most abundant precursor ions in the survey scan (300 to 1,600 Th). The resolution of the survey scan was 60,000 at *m/z* 200 Th, and for the MS/MS scan, the resolution was set to 15,000 at *m/z* 200 Th. The ion target value for the survey scans in the Orbitrap and the MS/MS scan were set to 3E6 and 1E5, respectively, and the maximum injection time was set to 60 ms for MS scan and for MS/MS scan. Parameters for acquiring HCD MS/MS spectra were a collision energy of 27 and an isolation width of 2.0 *m/z*. The precursors with unknown charge state and a charge state of 1 and 8 or greater than 8 were excluded. Peptides selected for MS/MS acquisition were then placed on an exclusion list for 20 s using the dynamic exclusion mode to limit duplicates.

Raw data were first submitted to Proteome Discoverer 2.2 using the SEQUEST HT search engine. A FASTA file composed of the OutB and OutD sequences was created. Precursor mass tolerance was set at 10 ppm, fragment mass tolerance was set at 0.02 Da, and up to 2 missed cleavages were allowed. The enzyme parameter was set to a combination of trypsin and chymotrypsin. Oxidation (Met), acetylation (protein N terminus), and the S^139^*p*BPA modification delta mass (+164.0626 Da) were set as variable modifications. Carbamidomethylation (Cys) was set as a fixed modification. Validation of the identified peptides was done by a “fixed-value” approach based on SEQUEST scores.

*p*BPA cross-linked peptides were mapped with the StavroX part of MeroX (2.0.1.4 version; http://stavrox.com). The following parameters were used: raw data converted in mgf format; site 1, Ser^139^BPA (noted as *x*); site 2, all amino acids with methionine oxidation as variable modification; precursor ion precision at 5 ppm and 0.02 Da on fragments. Validation of the candidates was based on a target-decoy analysis. A false discovery rate (FDR) was calculated for each candidate, and an FDR score cutoff was applied at the end.

### Chemical cross-linking and PG isolation.

Peptidoglycan was extracted from *D. dadantii* cells exponentially grown in BL broth supplemented with ampicillin and galacturonate. The cells (~5 × 10^10^) were washed with 50 mM sodium phosphate buffer (pH 7.2), resuspended in 2 mL of the same buffer, and added dropwise to 2 mL of boiling 10% SDS solution. After 1 h of boiling, the suspension was stored overnight at 30°C, and peptidoglycan was collected by centrifugation at 48,000 rpm in an SW 55 Ti rotor (Beckman Coulter) for 2 h at 30°C. Afterward, peptidoglycan was resuspended in 4 mL of 2% SDS solution, boiled again for 30 min, and collected by centrifugation at 48,000 rpm in an SW 55 Ti rotor for 1 h. Aliquots of purified peptidoglycan were boiled with Laemmli loading buffer containing 2-mercaptoethanol, and the eluted PG-bound proteins were analyzed by immunoblotting with anti-OutB and anti-OmpA antibodies.

### Immunoblotting.

The protein extracts were separated by electrophoresis on either 7.5, 9, 12 or 4 to 20% SDS-PAGE gels (homemade or from Bio-Rad), transferred to an Immobilon-P membrane (Millipore), and analyzed by immunoblotting with polyclonal rabbit antibodies raised against OutB, OutC, and OutD (39, 60), PelB and PemA (92), TolA (provided by J.C. Lazzaroni, MAP Lab), OmpA (generated against recombinant *D. dadantii* OmpA in this study), or PspA (provided by Hendrick Osadnik, Leibniz Universität Hannover) or monoclonal mouse Ro-LPS antibody (provided by J. C. Lazzaroni, MAP Lab).

### Enzymatic assays.

The plate assay for pectinase secretion was performed as described elsewhere (92). Bacteria were patched onto polygalacturonate-containing plates and grown at 30°C for 14 h. The plates were next flooded with a saturated solution of copper acetate to visualize the degradation halos. The halo size reflects the efficiency of pectinase secretion. For the immunoblotting secretion assay, *D. dadantii* strains carrying the appropriate plasmids were grown for 14 h at 28°C on LB broth supplemented with 0.2% galacturonate. Then, culture supernatants and cells were separated by SDS-PAGE and probed with antibodies raised against pectate lyase PelB and pectin methylesterase PemA. The ratio of the pectinases in culture supernatant reflects the efficiency of secretion. For β-galactosidase activity assays, E. coli MC3 carrying pREP4 and a CDBS-derived plasmid were grown in LB supplemented with ampicillin 100 μg/mL^−1^ and kanamycin 50 μg/mL^−1^ at 28°C for 5 h to an OD_600_ of ~0.5, induced with 1 mM IPTG, and grown for an additional 5 h. Afterward, the cells were permeabilized with toluene and 0.05% SDS and used in β-galactosidase assays, as described elsewhere (101).

### Cell fractionation.

The inner and outer membrane vesicles were separated on a discontinuous sucrose density gradient. E. coli MC3/pREP4 cells carrying a DBS-derived plasmid were grown in LB medium. One hundred milliliters of LB supplemented with ampicillin and kanamycin was inoculated with noninduced overnight culture to an OD_600_ of 0.1 and cultivated at 120 rpm and 28°C for 2 h; then, 1 mM IPTG was added, and culture was incubated for 7 h. Cells were collected, resuspended in 5 mL of 50 mM HEPES-NaOH (pH 7.4) containing protease inhibitor cocktail (Halt EDTA-free; Thermo), and disrupted with a French pressure cell. Unbroken cells and large debris were eliminated at 5,000 × *g* for 10 min. The cell extracts were supplemented with sucrose to 30% and loaded on a discontinuous 35%-to-65% sucrose gradient (with a 5% step). The gradients were centrifuged at 48,000 rpm in an SW 55 Ti rotor (Beckman Coulter) for 70 h; then, 0.3-mL fractions were collected from the bottom and analyzed by immunoblotting with the indicated antibodies. *D. dadantii* was grown on polygalacturonate-containing plates at 28°C for 36 h (3 plates per strain). The plate-grown cells were collected, washed with HEPES-NaOH (pH 7.4), broken with a French pressure cell, and separated as described above. When indicated, French pressure cell extracts were treated with lysozyme (0.1 mg/mL) at 25°C for 30 min prior to loading on a sucrose gradient.

### Phylogenetic analysis.

Candidate GspB homologs were searched at a protein level by using BLAST (102) and OutB HR (residues L127 to P191) as a query sequence against the Bacteria UniProtKB database. One thousand best hits were manually inspected to remove incomplete or fragment sequences, and 936 sequences were kept. Sequences were then clustered with CD-HIT software (103) with a 30% identity threshold to keep 118 representative sequence clusters. Multiple-sequence alignments were done with the T-Coffee program (104), and the HR domain sequences (corresponding to residues L127 to P191 of OutB) were selected and used for phylogenetic analyses. Phylogenetic analyses were performed, with the IQ tree web server in auto mode (105). A consensus tree was constructed from 1,000 bootstrap trees. The Robinson-Foulds distance between ML tree and consensus tree was 14. The phylogenetic tree was visualized with iTOL software (106).

### Data availability.

OutB structure coordinates have been deposited at the Protein Data Bank in Europe (PDBe) database with accession code 4WFW. The coordinates and structure factors are immediately available to the public.
