# Supplementary figures and images for: Scaffolding Protein GspB/OutB Facilitates Assembly of the Dickeya dadantii Type 2 Secretion System by Anchoring the Outer Membrane Secretin Pore to the Inner Membrane and to the Peptidoglycan Cell Wall
Source: mBio. 2022 May 12;13(3):e00253-22. doi: 10.1128/mbio.00253-22 (PMC9239104; doi:10.1128/mbio.00253-22)

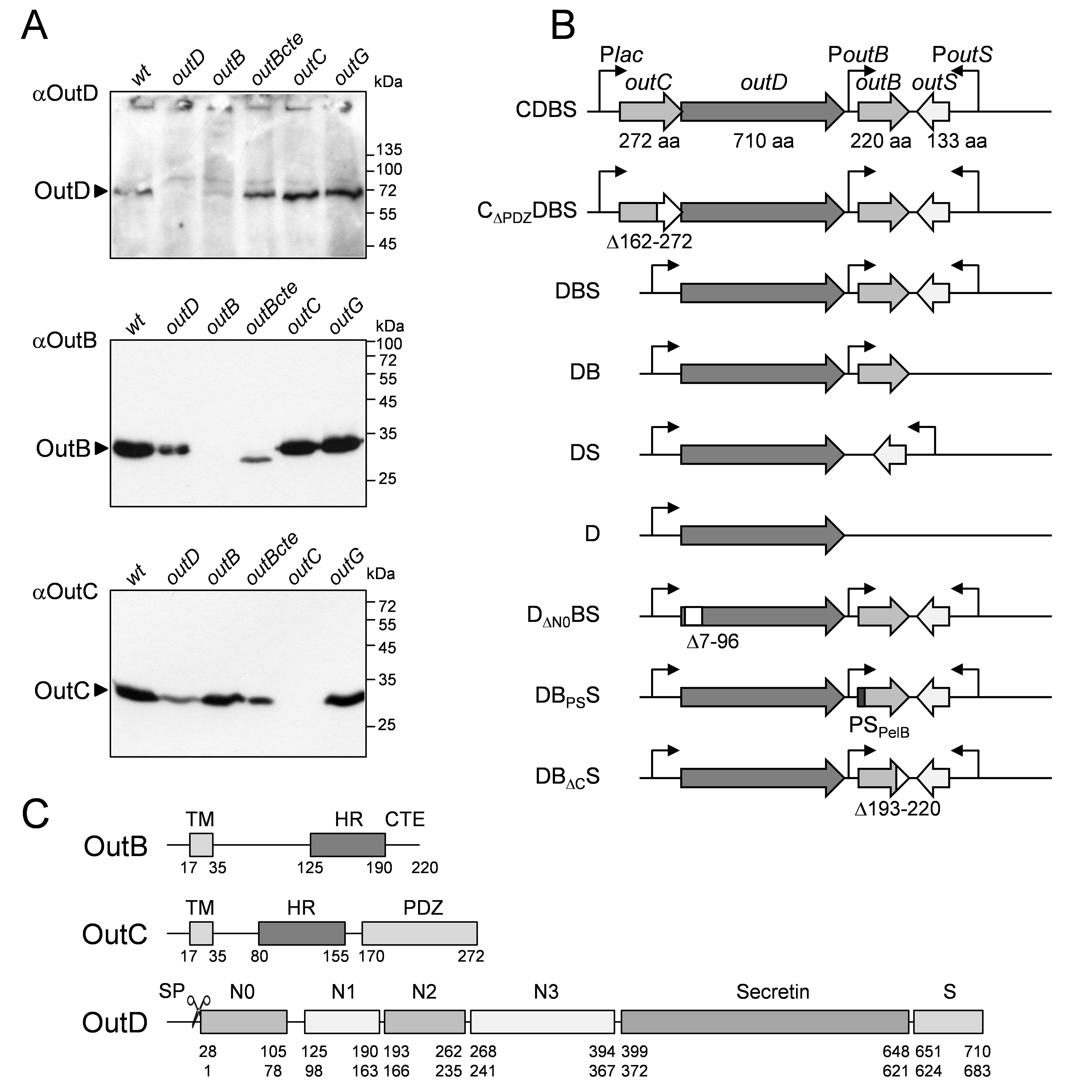

Supplement: FIG S1 [file mbio.00253-22-s0006.tif]

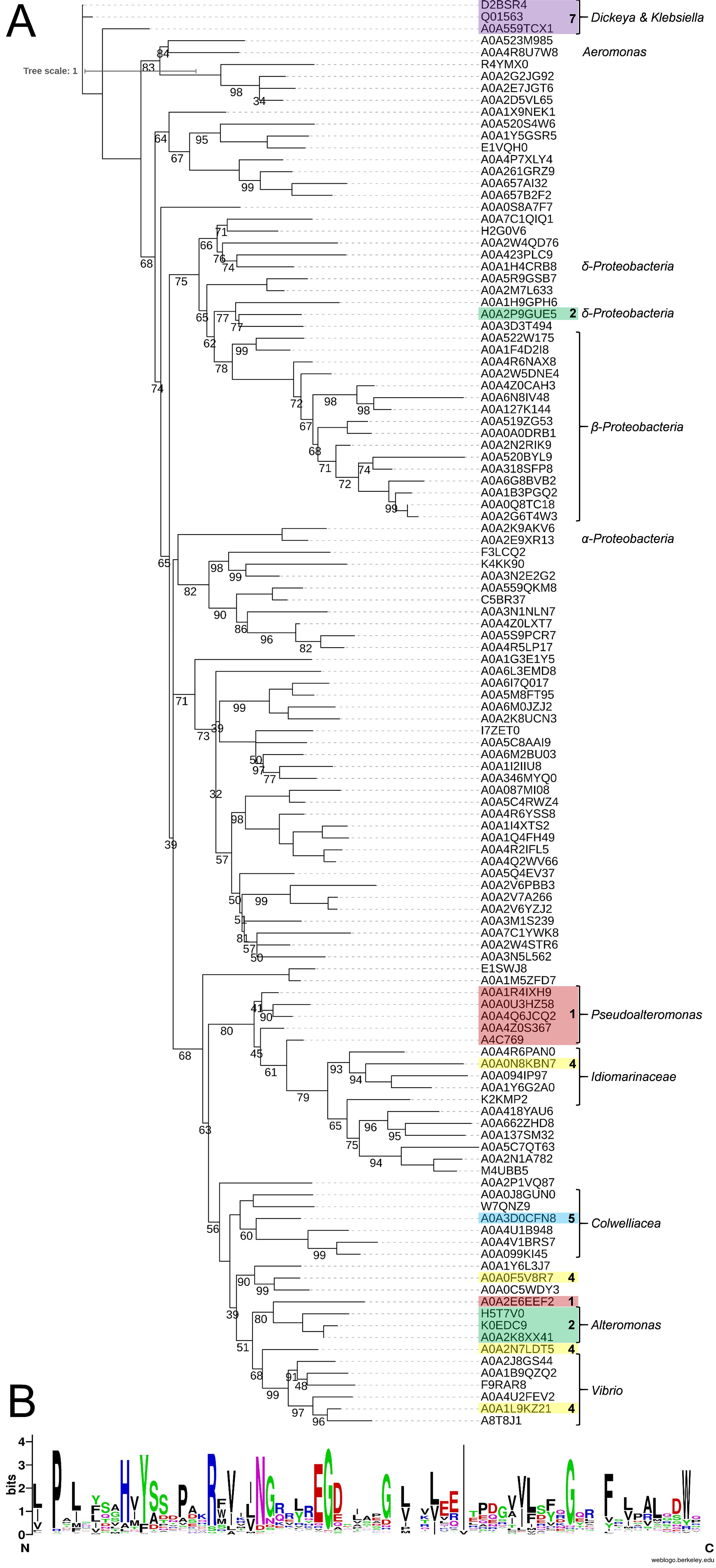

Supplement: FIG S8 [file mbio.00253-22-s0004.tif]

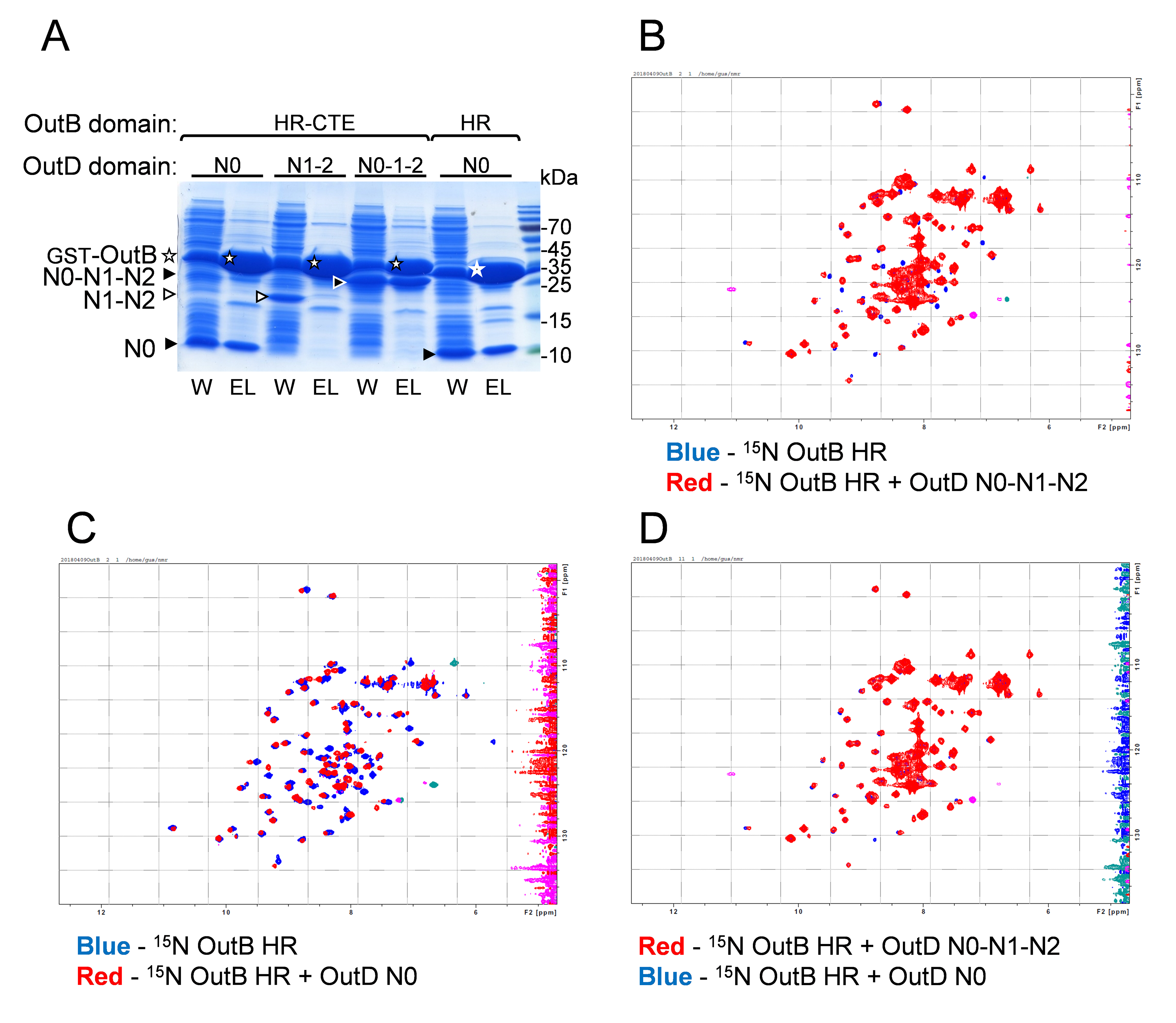

Supplement: FIG S2 [file mbio.00253-22-s0010.tif]

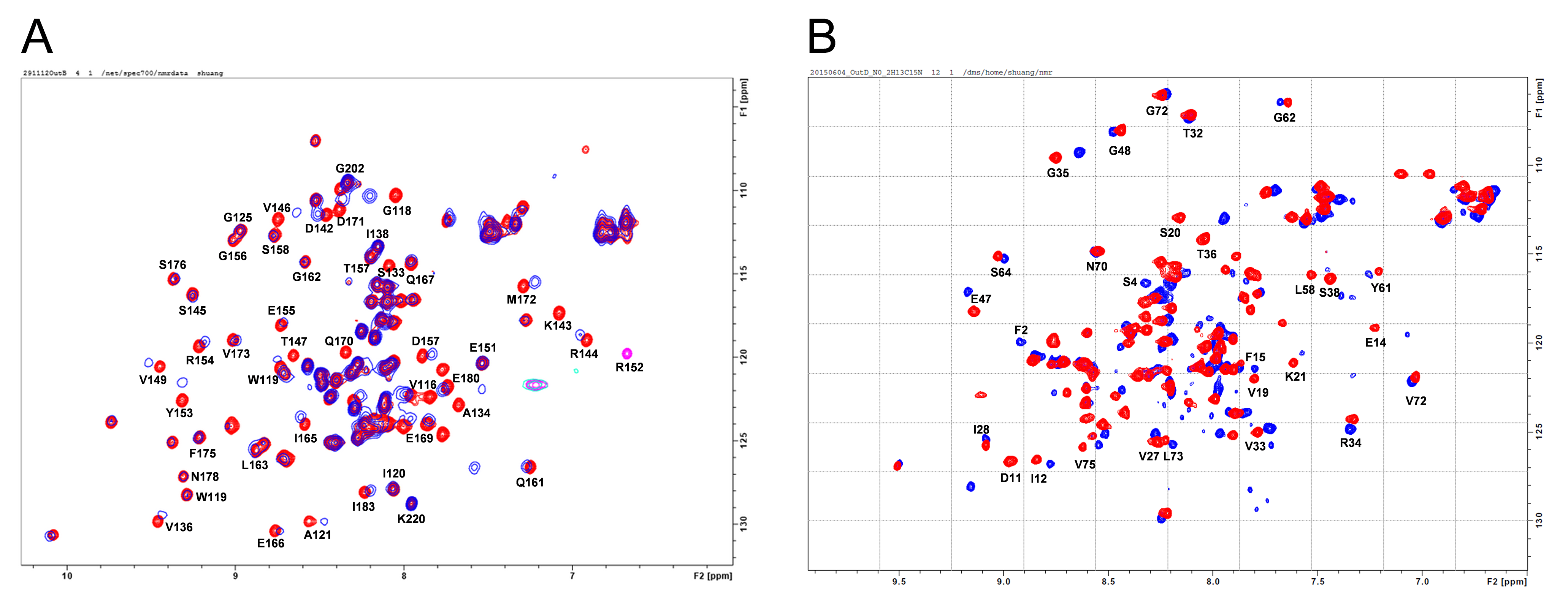

Supplement: FIG S3 [file mbio.00253-22-s0001.tif]

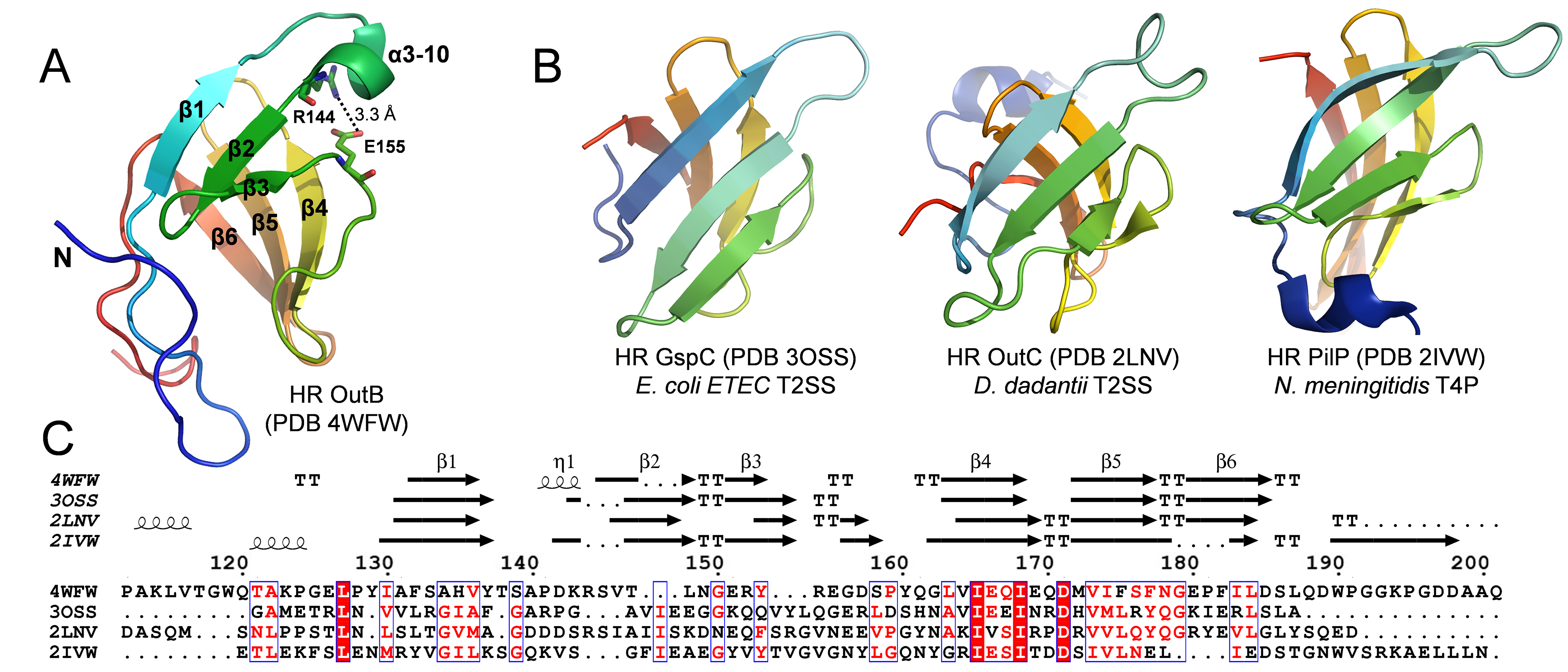

Supplement: FIG S4 [file mbio.00253-22-s0002.tif]

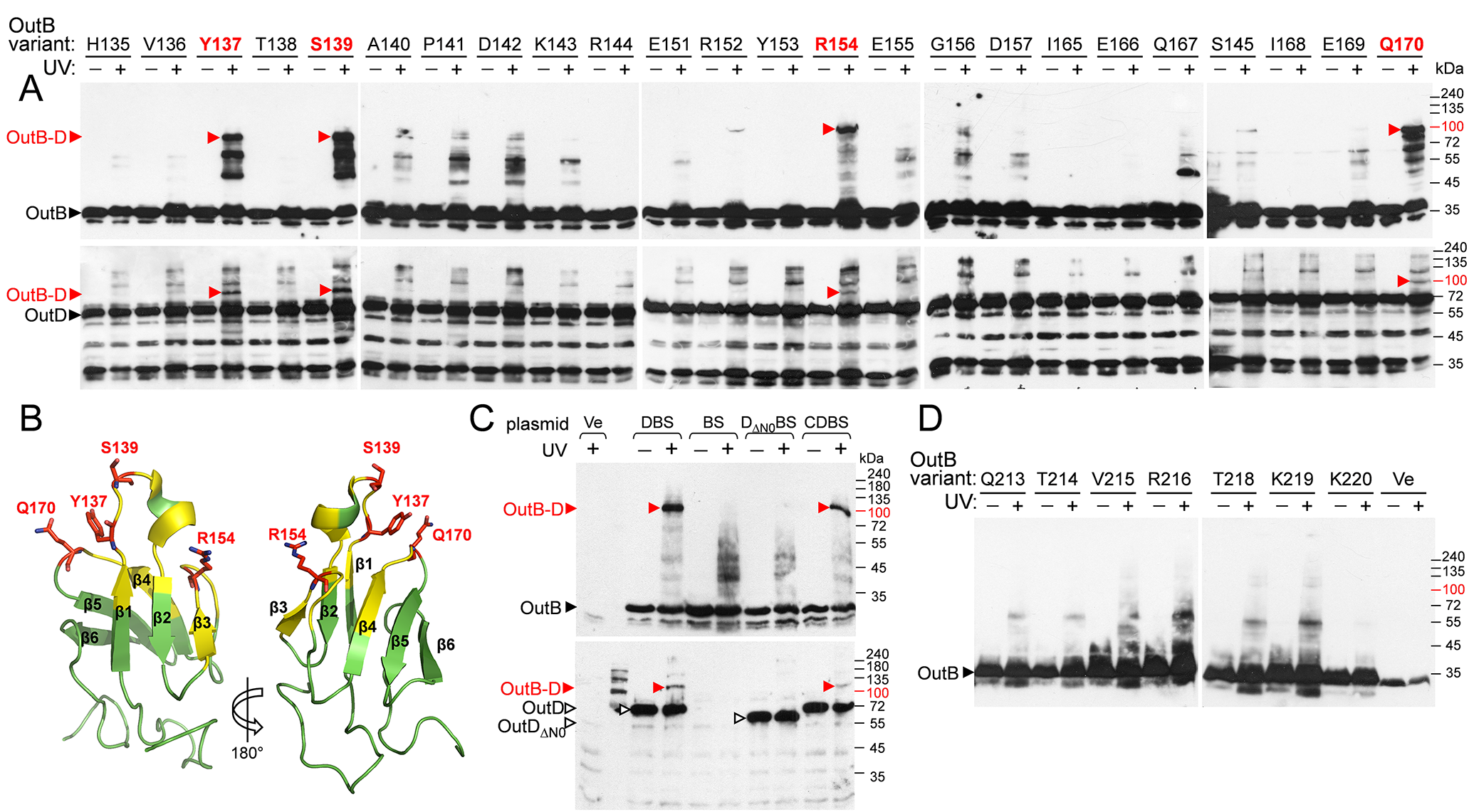

Supplement: FIG S5 [file mbio.00253-22-s0007.tif]

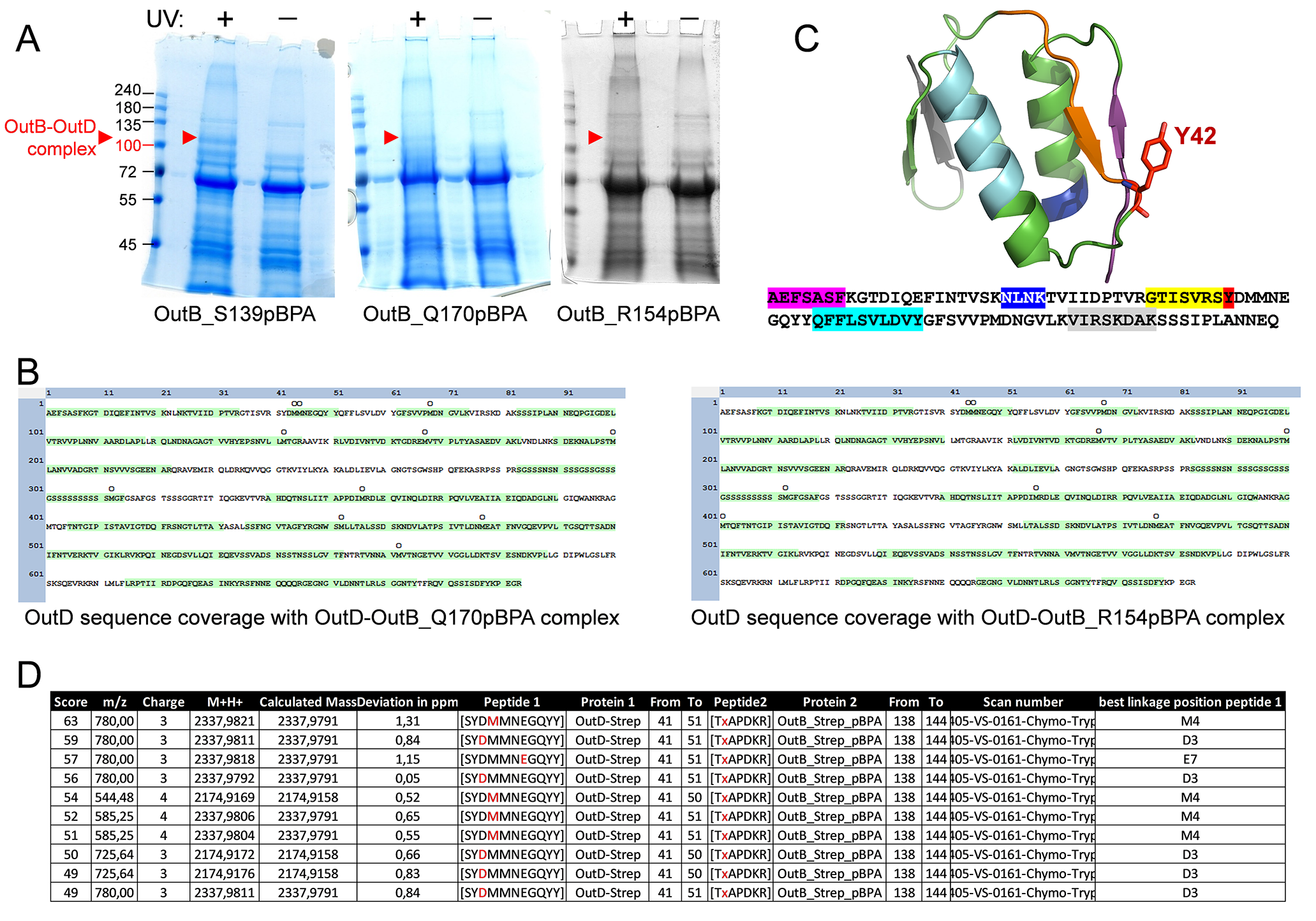

Supplement: FIG S6 [file mbio.00253-22-s0003.tif]

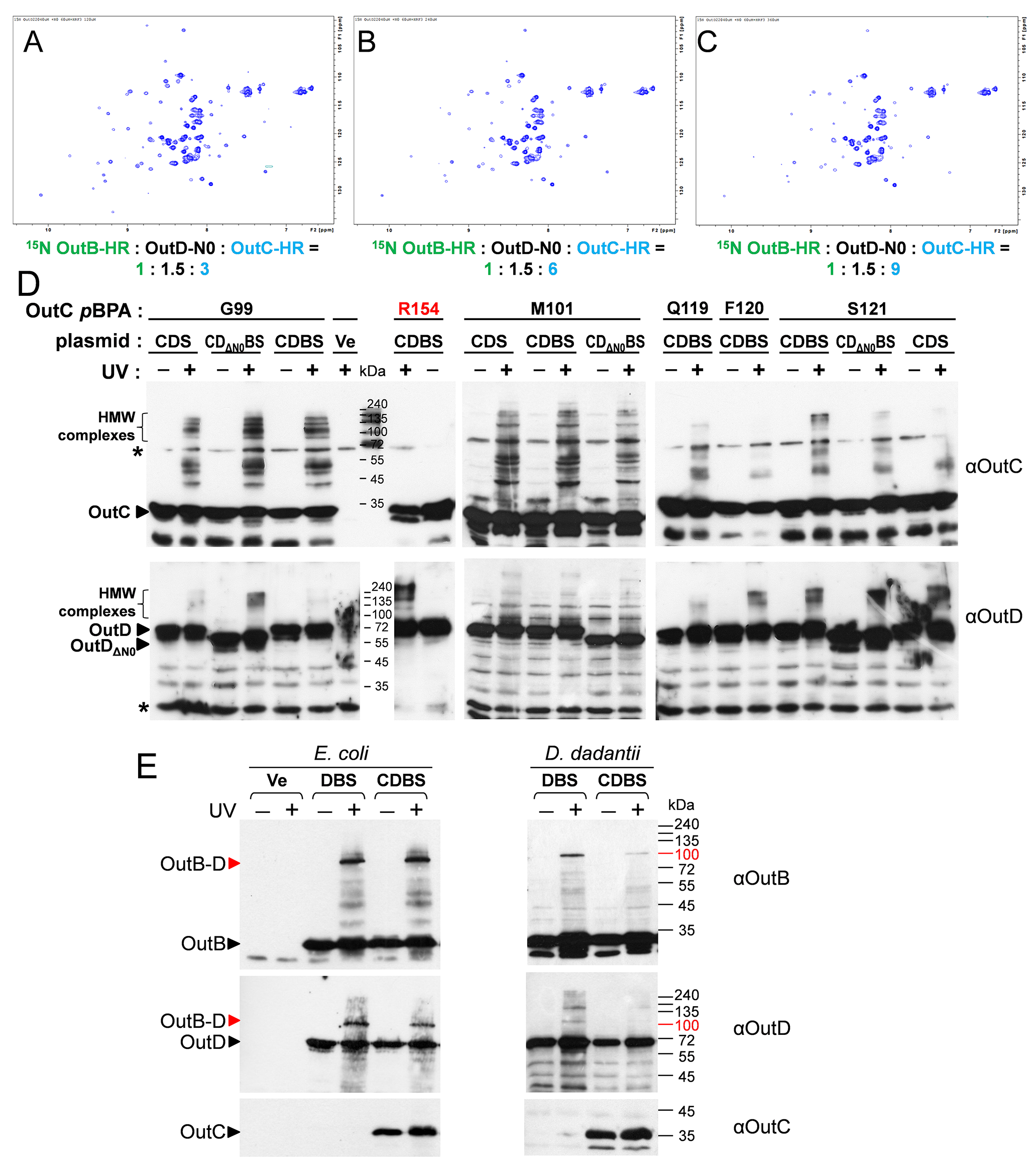

Supplement: FIG S7 [file mbio.00253-22-s0008.tif]
