# Supplementary material for: Scaffolding Protein GspB/OutB Facilitates Assembly of the Dickeya dadantii Type 2 Secretion System by Anchoring the Outer Membrane Secretin Pore to the Inner Membrane and to the Peptidoglycan Cell Wall
Source: mBio. 2022 May 12;13(3):e00253-22. doi: 10.1128/mbio.00253-22 (PMC9239104; doi:10.1128/mbio.00253-22)
Supplement: TABLE S2 [file mbio.00253-22-s0009.docx]

**Table S2. Bacterial strains, plasmids and primers used in this study**

________________________________________________________________________________

**Strain Genotype/phenotype Reference**

________________________________________________________________________________

*Escherichia coli*

BL21(DE3) F^–^ *dcm ompT* *hsdSB* (r_B_^–^, m_B_^–^) *gal lon λ* (DE3) Stratagene

NM522 *supE thi-1* ∆(*lac-proAB*) ∆(*mcrB-hsdSM*)*5* (*r*_K_^-^ *m*_K_^+^)

[F´ *proAB lacI*^q^*Z*∆*M15*] NEB

MG1655 K-12 F^–^ λ^–^ *ilvG*^–^ *rfb-50* *rph-1* laboratory collection

MC4100 F^−^*araD139*Δ(*argF*-*lac*)*U169 deoC1 flbB5301 ptsF25 rbsR*

*relA1 rpsL150* Casadaban, 1976

MC3 F^−^*araD139*Δ(*argF*-*lac*)*U169 deoC1 flbB5301 ptsF25 rbsR*

*relA1 rpsL150* λ[φ(*pspA::lacZ*)] Bergler *et al.*, 1994

*Dickeya dadantii* 3937

A3556 *lacZ2 prt*^−^ Δ*outC* Bouley *et al.,* 2001

A3558 *lacZ2 prt*^−^ Δ*outD* Bouley *et al.,* 2001

A5652 wild type laboratory collection

A5653 *outD::cat* (Cm^R^) This work

A5654 *outB::uidA-nptI* (Km^R^) This work

A5719 *outB::cat* (Cm^R^) This work

A5916 outB::*nptI-sacB-sacR* (Km^R^) This work

A5954 *outB*Δ*cte* This work

A6078 *outB::tetA* (Tc^R^) This work

A6533 Δ*outD* (N117-R648 non-phase deletion) This work

________________________________________________________________________________

__________________________________________________________________________________________

**Plasmid Genotype/phenotype Reference**

__________________________________________________________________________________________

pGEM-T P*lac*, P*T7pol*, *blaM* (Ap^R^) Promega

pET-20b(+) P*T7pol*, coding PelB signal peptide and 6His, *blaM* (Ap^R^) Novagen

pSup-BpaRS-6TRN (pSup) amber suppressor tRNA and aminoacyl-tRNA synthetase

for incorporation of *p*BPA, *cat* (Cm^R^) Ryu & Schultz, 2006

pREP4 *lacI^q^*, *neo* (Km^R^) Qiagen

pGEX-6P-3 P*lac*, coding cleavable N-terminal GST, *blaM* (Ap^R^) GE (Helthcare)

CDBS ^a^ pGM-T carrying *outC*, *outD*, *outB* and *outS* This work

DBS ^a^ pGM-T carrying *outD*, *outB* and *outS* This work

DB ^a^ pGM-T carrying *outD* and *outB* This work

DS ^a^ pGM-T carrying *outD* and *outS* This work

D ^a^ pGM-T carrying *outD* This work

pGX-oB_HR-CTE_ pGEX-6P-3 carrying *GST-outB* (aa P112 to K220) This work

pGX-oB_HR_ pGEX-6P-3 carrying *GST-outB* (aa P112 to G192) This work

pGX-oB_HR2_ pGEX-6P-3 carrying *GST-outB* (aa P112 to G202) This work

pGX-oB_HR-CTE_-oD_N0-N1-N2_ pGEX-6P-3 carrying *GST-outB* (aa P112 to K220) followed

by *N0-N1-N2-outD-6His* (aa A1 to V258) ^b^ This work

pGX-oB_HR-CTE_-oD_N0_ pGEX-6P-3 carrying *GST-outB* (aa P112 to K220) followed

by *N0-outD-6His* (aa A1 to S85) ^b^ This work

pGX-oB_HR-CTE_-oD_N1-N2_ pGEX-6P-3 carrying *GST-outB* (aa P112 to K220) followed

by *N1-N2-outD-6His* (aa A89 to V258) ^b^ This work

pGX-oB_HR_-oD_N0_ pGEX-6P-3 carrying *GST-outB* (aa P112 to G192) followed

by *N0-outD-6His* (aa A1 to S85) ^b^ This work

pET-oD28-112 pET-20b carrying *N0-outD-6His* (aa A1 to S85) ^b^ Login *et al.*, 2010

pET-oD28-285 pET-20b carrying *N0-N1-N2-outD-6His* (aa A1 to V258) ^b^ Login *et al.*, 2010

________________________________________________________________________________________

^a^ More details about these constructs are given on Fig. S1B.

^b^ Residue numbering is this for the matured, signal peptide-less OutD

__________________________________________________________________________________

**Primer Nucleotide sequence (5’-3’)^b^ Generated mutation^c^**

__________________________________________________________________________________

OutB-BH-5’ **ctgggatcc**cccgccaaattggtaacag

OutB-XB-RI-3’ **cggaattccggctctaga**gcatgatgtgcagttgctg

OuB_H135TAG^a^ gtatatcgccttcagcgcg**t**a**g**gtctatacgtctgctccg HR(OutB)H135tag

OuB_V136TAG^a^ cgccttcagcgcgcat**tag**tatacgtctgctccggac HR(OutB)V136tag

OuB_Y137TAG^a^ ccttcagcgcgcatgtcta**g**acgtctgctccggacaag HR(OutB)Y137tag

OuB_T138TAG^a^ cttcagcgcgcatgtctat**ta**gtctgctccggacaagcg HR(OutB)T138tag

OuB_S139TAG^a^ gcgcgcatgtctatacgt**ag**gctccggacaagcgcagc HR(OutB)S139tag

OuB_A140TAG^a^ gcgcatgtctatacgtct**tag**ccggacaagcgcagcgttac HR(OutB)A140tag

OuB_P141TAG^a^ gcatgtctatacgtctgct**ta**ggacaagcgcagcgttacc HR(OutB)P141tag

OuB_D142TAG^a^ gtctatacgtctgctccg**t**a**g**aagcgcagcgttaccctg HR(OutB)D142tag

OuB_K143TAG^a^ ctatacgtctgctccggac**t**agcgcagcgttaccctgaac HR(OutB)K143tag

OuB_R144TAG^a^ cgtctgctccggacaag**tag**agcgttaccctgaacggag HR(OutB)R144tag

OuB_S145TAG^a^ ctgctccggacaagcgc**tag**gttaccctgaacggagag HR(OutB)S145tag

OuB_E151TAG^a^ gcgttaccctgaacgga**t**agcgctaccgtgaaggc HR(OutB)E151tag

OuB_R152TAG^a^ gttaccctgaacggagag**tag**taccgtgaaggcgacagc HR(OutB)R152tag

OuB_Y153TAG^a^ ctgaacggagagcgcta**g**cgtgaaggcgacagccc HR(OutB)Y153tag

OuB_R154TAG^a^ ctgaacggagagcgctac**tag**gaaggcgacagcccgtatc HR(OutB)R154tag

OuB_E155TAG^a^ gaacggagagcgctaccgt**t**a**g**ggcgacagcccgtatcag HR(OutB)E155tag

OuB_G156TAG^a^ ggagagcgctaccgtgaa**tag**gacagcccgtatcagggg HR(OutB)G156tag

OuB_D157TAG^a^ gagcgctaccgtgaaggc**tag**agcccgtatcaggggttg HR(OutB)D157tag

OuB_I165TAG^a^ ccgtatcaggggttggtg**tag**gagcagattgagcaggat HR(OutB)I165tag

OuB_E166TAG^a^ gtatcaggggttggtgatc**t**agcagattgagcaggatatg HR(OutB)E135tag

OuB_Q167TAG^a^ caggggttggtgatcgag**t**agattgagcaggatatgg HR(OutB)Q135tag

OuB_I168TAG^a^ ggggttggtgatcgagcag**tag**gagcaggatatggtgatc HR(OutB)I135tag

OuB_E169TAG^a^ gttggtgatcgagcagatt**t**agcaggatatggtgatcttc HR(OutB)E135tag

OuB_Q170TAG^a^ gtgatcgagcagattgag**t**aggatatggtgatcttcag HR(OutB)Q170tag

OuB_G193TGA^a^ gttgcaggattggccgggc**t**g**a**aaaccgggcgatgacgcc HR(OutB)G193tga

OuB_Q213TAG^a^ ccgacgtcaaaaccggag**t**a**g**accgtcaggacaacgaag CTE(OutB)Q213tag

OuB_T214TAG^a^ cgtcaaaaccggagcaa**tag**gtcaggacaacgaagaaatg CTE(OutB)T214tag

OuB_V215TAG^a^ caaaaccggagcaaacc**tag**aggacaacgaagaaatgac CTE(OutB)V215tag

OuB_R216TAG^a^ ccggagcaaaccgtc**ta**gacaacgaagaaatgac CTE(OutB)R216tag

OuB_T218TAG^a^ gagcaaaccgtcaggaca**ta**gaagaaatgacacagc CTE(OutB)T214tag

OuB_K219TAG^a^ caaaccgtcaggacaacg**t**agaaatgacacagcaac CTE(OutB)K219tag

OuB_K219TGA^a^ gcaaaccgtcaggacaacg**tga**aaatgacacagcaactgcac CTE(OutB)K219tga

OuB_Y137C^a^ ccttcagcgcgcatgtct**gc**acgtctgctccggacaagc HR(OutB)Y137C

OuB_T138C^a^ cttcagcgcgcatgtctat**tgc**tctgctccggacaagcgc HR(OutB)T138C

OuB_S139C^a^ gcgcgcatgtctatacg*t****gc***gctccggacaagcgcagc HR(OutB)S139C

OuB_Y153C^a^ cctgaacggagagcgct**g**ccgtgaaggcgacagcc HR(OutB)Y153C

OuB_R154C^a^ ctgaacggagagcgctac**t**gtgaaggcgacagcccg HR(OutB)R154C

OuC_G99TAG^a^ cgctgaacctttcgctgacc**tag**gtcatggccggcgatgacg HR(OutC)G99tag

OuC_M101TAG^a^ cctttcgctgaccggcgtc**ta**ggccggcgatgacgattcccg HR(OutC)M101tag

OuC_Q119TAG^a^ catcagtaaagacaacgaa***t****ag*ttcagtcgtggcgtcaatg HR(OutC)Q119tag

OuC_F120TAG^a^ cagtaaagacaacgaacag*t****ag***agtcgtggcgtcaatgaag HR(OutC)F120tag

OuC_S121TAG^a^ cagtaaagacaacgaacagttc***tag***cgtggcgtcaatgaagagg HR(OutC)S121tag

OuC_S162tga aggat**tga**gggtcggacggcgttcctggc ΔPDZ (OutC) ΔS162-E272

ROuC_S162tga gaccc**tca**atcctcctggctgtacaatcc ΔPDZ (OutC) ΔS162-E272

OuD_E29TAG^a^ ccgtgtggctgggctgcc**t**a**g**ttttcagccagtttcaaagg N0(OutD)E2tag

OuD_F30TAG^a^ gtggctgggctgccgaat**ag**tcagccagtttcaaaggaac N0(OutD)F3tag

OuD_S31TAG^a^ ggctgggctgccgaatttt**ag**gccagtttcaaaggaacc N0(OutD)S4tag

OuD_A32TAG^a^ ctgggctgccgaattttca**tag**agtttcaaaggaaccgata N0(OutD)A5tag

OuD_S33TAG^a^ ggctgccgaattttcagcc**tag**ttcaaaggaaccgatattc N0(OutD)S6tag

OuD_F34TAG^a^ gccgaattttcagccagtt**ag**aaaggaaccgatattcagg N0(OutD)F7tag

OuD_R67TAG^a^ cgcggcaccatcagcgtg**tag**agttacgacatgatgaacg N0(OutD)R40tag

OuD_S68TAG^a^ ggcaccatcagcgtgcgc**tag**tacgacatgatgaacgaag N0(OutD)S41tag

OuD_Y69TAG^a^ ccatcagcgtgcgcagtta**g**gacatgatgaacgaaggg N0(OutD)Y42tag

OuD_Y69C^a^ ccatcagcgtgcgcagtt**g**cgacatgatgaacgaaggg N0(OutD)Y42C

OutDStrep gccggcaacggaaccagcggc**tggagccacccgcagttcgaaaaa** N3(OutD)WSHPQFEK

ROutDStrep gggcgaggacggccgcgacgc**tttttcgaactgcgggtggctcca** N3(OutD)WSHPQFEK

5OuDdltN0 **attttcagcc**ggtgatga gctggtgaccc N0(OutD) ΔF7-I96

3OuDdltN0 ctcatcaccggctgaaaa**ttcggcagcc** N0(OutD) ΔF7-I96

_______________________________________________________________________________

^a^ For each primers used in site directed mutagenesis, another primer with reverse complementary sequence was used (not shown).

^b^ Mutated or introduced bases are in bold.

^c^ Residue numbering is this for the matured, signal peptide-less OutD.

**Table S3. GspB representatives used in the sequence alignment on Fig. 2A**

| Short name ^a^ | Species | Accession number |
| --- | --- | --- |
| OutB_Dd | *Dickeya dadantii* (strain 3937) | Q01563 |
| OutB_Ds | *Dickeya solani* | A0A2K8VW55 |
| OutB_Dz | *Dickeya zeae* | D2BSR4 |
| OutB_Da | Dickeya aquatica | A0A375A847 |
| OutB_Dc | Dickeya paradisiaca | WP_023638232 |
| GspB_Lb | *Lonsdalea britannica* | A0A1X3RVC9 |
| OutB_Pa | *Pectobacterium atrosepticum* | Q6D2I2 |
| OutB_Pc | *Pectobacterium carotovorum* | C6DAQ9 |
| PulB_Ko | *Klebsiella oxytoca* | A0A0H3H3J5 |
| PulB_Kp | *Klebsiella pneumoniae* | W9BIG8 |
| ExeB_As | *Aeromonas salmonicida* | A4SIF9 |
| ExeB_Ah | *Aeromonas hydrophila* | A0KPN3 |
| EpsB_Vp | *Vibrio parahaemolyticus* | Q87SK7 |
| EpsB_Vc | *Vibrio cholerae* | Q9KPC8 |
