# Supplementary material for: Scaffolding Protein GspB/OutB Facilitates Assembly of the Dickeya dadantii Type 2 Secretion System by Anchoring the Outer Membrane Secretin Pore to the Inner Membrane and to the Peptidoglycan Cell Wall
Source: mBio. 2022 May 12;13(3):e00253-22. doi: 10.1128/mbio.00253-22 (PMC9239104; doi:10.1128/mbio.00253-22)
Supplement: TABLE S1 [file mbio.00253-22-s0005.docx]

**Table S1. Data collection and refinement statistics**

| **Data collection** | |
| --- | --- |
| Space group | P 4_3_ 2_1_ 2 |
| Cell parameters (Å/°) | a=b=36.3, c=133.2 α=β=γ=90 |
| Molecules per asymmetric unit | 1 |
| Wavelength (Å) | 1.07 |
| Resolution (Å) | 2.02-36.30 (2.02-2.08) |
| Total number of observations | 56965 (2403) |
| Number of unique reflections | 6380 (435) |
| Multiplicity | 8.9 (5.5) |
| Completeness (%) | 99.2 (93.6) |
| R_merge_ (%)^a^ | 0.036 (0.574) |
| Mean <I/σ(I)> | 30.6 (3.7) |
| Wilson B-factor (Å^2^) | 44.4 |
| R_pim_(I) | 0.014 (0.280) |
| R_meas_ | 0.041 (0.690) |
| **Refinement** | |
| Resolution limits (Å) | 2.05-34.91 |
| R-factor (%) / R-free^b^ (%) | 20.85 / 25.42 |
| RMSD bonds (Å)/RMSD angle (°) | 0.015/1.81 |
| Average B-factor (Å^2^) | 33.4 |
| Number of protein atoms | 666 |
| Number of solvent atoms | 15 |
| **Ramachandran plot statistics** | |
| Residues in most favoured regions (%) | 100 |
| Residues in additional allowed regions (%) | 0 |

The parameter values for the highest resolution shell are given in parentheses. The values presented in this Table are from SCALA (Evans 2006) and REFMAC from the CCP4 suite.

^a^R_merge_ = Σ_hkl_ Σ_i_ |I_i_ - <I> | /-Σ_hkl_ ΣI_i_, where I_i_ is the intensity of the i^th^ observation, <I> is the mean intensity of the reflection and the summations extend over all unique reflections (hkl) and all equivalents (i), respectively. ^b^R-factor = Σ_hkl_ |F_o_ – F_c_ | / Σ_hkl_ F_o_, where F_o_ and F_c_ represent the observed and calculated structure factors, respectively. The R-Factor is calculated using 90% of the data included in refinement and R-free the 10% excluded.
